# Cryo-EM structure of the brine shrimp mitochondrial ATP synthase suggests an inactivation mechanism for the ATP synthase leak channel

**DOI:** 10.1038/s41418-025-01476-w

**Published:** 2025-03-19

**Authors:** Amrendra Kumar, Juliana da Fonseca Rezende e Mello, Yangyu Wu, Daniel Morris, Ikram Mezghani, Erin Smith, Stephane Rombauts, Peter Bossier, Juno Krahn, Fred J. Sigworth, Nelli Mnatsakanyan

**Affiliations:** 1 Department of Cell and Biological Systems, Penn State College of Medicine, Hershey, PA USA; 2 Department of Cellular and Molecular Physiology, Yale University School of Medicine, New Haven, CT USA; 3 Department of Plant Biotechnology and Bioinformatics, Ghent University, Ghent, Belgium; 4 VIB Center for Plant Systems Biology, VIB, Ghent, Belgium; 5 Faculty of Bioscience Engineering, Ghent University, Ghent, Belgium; 6 National Institute of Environmental Health Sciences, Durham, NC USA

**Keywords:** Cryoelectron microscopy, Calcium channels

## Abstract

Mammalian mitochondria undergo Ca^2+^-induced and cyclosporinA (CsA)-regulated permeability transition (mPT) by activating the mitochondrial permeability transition pore (mPTP) situated in mitochondrial inner membranes. Ca^2+^-induced prolonged openings of mPTP under certain pathological conditions result in mitochondrial swelling and rupture of the outer membrane, leading to mitochondrial dysfunction and cell death. While the exact molecular composition and structure of mPTP remain unknown, mammalian ATP synthase was reported to form voltage and Ca^2+^-activated leak channels involved in mPT. Unlike in mammals, mitochondria of the crustacean *Artemia franciscana* have the ability to accumulate large amounts of Ca^2+^ without undergoing the mPT. Here, we performed structural and functional analysis of *A. franciscana* ATP synthase to study the molecular mechanism of mPTP inhibition in this organism. We found that the channel formed by the *A. franciscana* ATP synthase dwells predominantly in its inactive state and is insensitive to Ca^2+^, in contrast to porcine heart ATP synthase. Single-particle cryo-electron microscopy (cryo-EM) analysis revealed distinct structural features in *A. franciscana* ATP synthase compared with mammals. The stronger density of the e-subunit C-terminal region and its enhanced interaction with the c-ring were found in *A. franciscana* ATP synthase. These data suggest an inactivation mechanism of the ATP synthase leak channel and its possible contribution to the lack of mPT in this organism.

## Introduction

Mitochondrial permeability transition (mPT) is a phenomenon discovered in mammals several decades ago [1–4]. It is mediated by the opening of the Ca^2+^-regulated ion channel in the inner mitochondrial membrane, called the mitochondrial permeability transition pore (mPTP) or mitochondrial megachannel (MMC). mPTP activation allows the flux of ions and molecules with a molecular weight of up to 1.5 kDa in and out of the mitochondrial matrix. It can cause transient or prolonged depolarization of mitochondrial membrane potential [3, 4]. The brief or transient openings of mPTP serve its physiological function to regulate embryonic development, calcium handling, and metabolism [5, 6]. In contrast, prolonged openings induce irreversible changes in mitochondrial morphology and function, leading to cell death. mPTP is reported to play a critical role in different pathologies, such as ischemia/reperfusion injury of the heart and brain, Alzheimer’s and Parkinson’s diseases and Amyotrophic Lateral Sclerosis (ALS) [7–10].

Despite extensive research, the exact molecular composition of mPTP and its gating mechanism remains elusive. Over the years, different mitochondrial proteins, including the voltage-dependent anion channel (VDAC) [11, 12], the phosphate carrier (PiC) [13], the translocator protein (TSPO) [14–16], and the adenine nucleotide translocator (ANT) [17] were reported to be the structural component of mPTP. Nevertheless, genetic ablation of these proteins revealed their role as regulators of the pore rather than the channel-forming elements of mPTP [18–21]. Patch-clamp recordings of mitoplasts (inner mitochondrial membrane preparations) were used for the biophysical characterization of mPTP. The mPTP forms voltage-gated, Ca^2+^ and cyclophilin D (CypD)-regulated, non-selective high-conductance channels with 1.5 nS peak conductance activity. *Bos taurus* (bovine) and *Sus scrofa* (porcine) ATP synthase dimers [22–26] and monomers [27–33] were reported to have similar properties to mPTP in several recent studies [22, 27, 29, 32–35]. The ATP synthase c-subunit ring was suggested to comprise the structural pore-forming component of mPTP [27–33, 35]. The channel formed by the c-ring, termed ATP synthase c-subunit leak channel (ACLC), is a multi-conductance, non-selective, voltage-gated, Ca^2+^-sensitive channel with 1.5 nS peak conductance activity [32, 35]. The Ca^2+^-binding sites were found at the β-subunits of ATP synthase, and application of Ca^2+^ during patch-clamp recordings of mammalian ATP synthase activated the channel [34]. The application of purified CypD was also reported to activate the channel during electrophysiology recordings of ATP synthase, while its binding partner, cyclosporin A (CsA), inhibited it [32].

Purified human ATP synthase c-subunit ring alone formed high-conductance channels during electrophysiology recordings [32, 35]. Its activity was inhibited upon the addition of ATP synthase F_1_ domain, suggesting F_1_ constitutes the inactivation gate and regulator of the channel from the matrix side [35]. Mitochondria isolated from the ATP synthase c-subunit knockout HAP1-A12 cells were shown to lack the 1.5 nS high conductance activity, as compared to WT, during patch-clamp experiments [28]. A low conductance channel activity, sensitive to ANT inhibitor bongkrekic acid (BA), was still present in these knockout cells, suggesting the possible contribution of multiple pores to mPT [28]. Liver mitochondria from mice with triple deletion of *Ant1*, *Ant2*, and *Ant4* still undergo a CsA-sensitive mPT at increased matrix Ca^2+^ overload [36]. The deletion of *Ppif* gene encoding CypD in these mice completely prevented Ca^2+^-induced mPT, suggesting a new model consisting of two distinct molecular components, ANT and another CypD-regulated channel, that mediate mPTP formation [36]. Several other studies reported the potential existence of a transient, “non-conventional” form of mPT in mammals insensitive to CsA and not regulated by CypD [37–39]. Thus, the exact molecular nature of mPTP in mammals is still being debated and remains to be elucidated.

In contrast to mammals, the embryos of the crustacean brine shrimp *A. franciscana* maintain viability for several years under anoxic diapause conditions without undergoing mPT [40–43]. The ability of *A. franciscana* to tolerate environmental insults can be explained by the lack of Ca^2+^-regulated mPTP in this organism. No swelling of the mitochondrial matrix, rupture of the outer membrane, or cytochrome *c* release were observed in *A. franciscana* mitochondria upon profound calcium storage [40]. Yet, the underlying molecular mechanism of mPTP inhibition in this crustacean is not fully understood.

This study investigated the structure and leak channel activity of *A. franciscana* ATP synthase and its possible contribution to Ca^2+^-resistant mPT. Our data show that *A. franciscana* ATP synthase forms channels with significantly different channel properties when compared with the porcine heart ATP synthase. *A. franciscana* ATP synthase demonstrated transient, Ca^2+^-insensitive openings after prolonged inactivity in planar lipid bilayer recordings. Single-particle cryo-electron microscopy analysis of *A. franciscana* ATP synthase revealed a similar subunit composition and organization to mammalian ATP synthases [44–47]. It consists of two molecular domains, soluble F_1_ and membrane-embedded F_O_. Despite the observed similarities, *A. franciscana* ATP synthase structure had substantial differences. All three α-subunits (α_TP_, α_DP_, α_E_) had strong densities of N-terminal alpha-helical segments and enhanced interactions with the OSCP, compared with the mammalian structures, where at least one out of the three α-subunits lack this density [46–49].

Furthermore, the C-terminal region of the F_O_ e-subunit had a stronger density in the *A. franciscana* ATP synthase cryo-EM maps compared with all known mammalian structures [46–48]. In addition, the e-subunit was found to be longer by 14 amino acid residues and had a more curved C-terminal region shifted towards the c-ring. The proximity of the e-subunit C-terminus to the c-ring may favor interactions with the N-terminal residues of the ring and lipids occupying its cavity. A peptide with the corresponding sequence of the *A. franciscana* e-subunit C-terminal region inhibited the human c-subunit channel activity during electrophysiology recordings. These findings suggest that the e-subunit may form an inactivation gate of the c-subunit channel acting from the intermembrane space in concert with F_1_, which acts at the matrix side. Distinct structural features of the *A. franciscana* e-subunit may lead to enhanced interactions with the c-ring and contribute to the inactivation of the ATP synthase c-subunit leak channel in this organism.

## Results

### Mitochondria isolated from *A. franciscana* lack Ca^2+^-induced and cyclosporin A-sensitive mitochondrial permeability transition

**Fig. 1:**
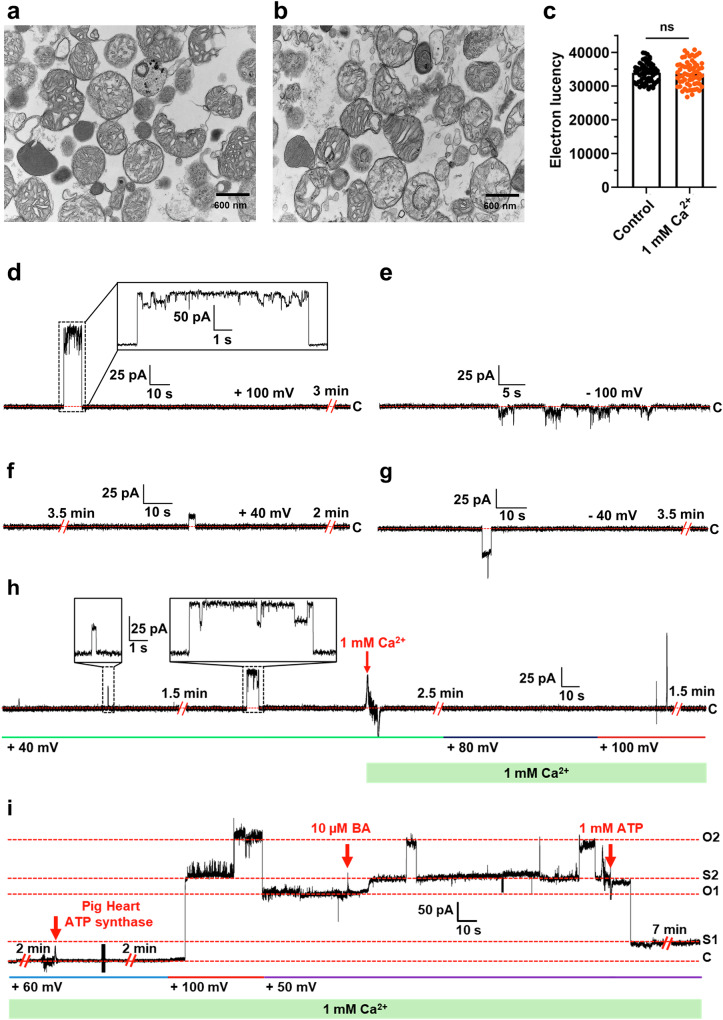
*A. franciscana* mitochondria lack the Ca^2+^-induced mitochondrial permeability transition and Ca^2+^-sensitive large conductance channel of ATP synthase. **a** EM images of *A. franciscana* mitochondria before and **b** after treatment with 500 μM Ca^2+^ for 20 min before they were fixed for further EM analysis. **c** Group data showing no change in electron lucencies before and after treatment with Ca^2+^ (unpaired *t*-test was used). **d**–**g** Representative single-channel recordings of *A. franciscana* ATP synthase (purified at pH 7.0) at +100 mV, –100 mV, +40 mV, –40 mV, respectively, showing that the channel mainly dwells in its inactive, closed state and forms brief openings. **h** Representative continuous single-channel recording of *A. franciscana* ATP synthase. The channel continued to remain closed or have brief, infrequent openings after the addition of 1 mM Ca^2+^. **i** Representative continuous single-channel recording of porcine ATP synthase in the presence of 1 mM Ca^2+^, showing characteristic large-conductance channel activity and sensitivity to ATP (1 mM). Bongkrekic acid (BA) (10 μM) was added to confirm that the ANT channels are not responsible for the recorded currents due to possible contamination. C refers to the closed state of the channel, O refers to the open state of the channel, and S1 and S2 refer to the sub-conductance states. The green boxes in (**h**) and (**i**) indicate the presence of 1 mM Ca^2+^ during the recording. Signals were filtered at 5 kHz using the amplifier circuitry.

Mitochondria isolated from *A. franciscana* were reported to sequester large amounts of calcium with no evidence of swelling, rupture of the inner and outer membranes, and activation of mPTP [40, 41], unlike mammalian mitochondria [1, 2]. We performed the transmission electron microscopy imaging on *A. franciscana* mitochondria, which further confirmed the lack of swelling of the mitochondrial matrix upon Ca^2+^ treatment (Fig. 1a–c).

The calcium retention capacity experiment (CRC) is a widely used method to quantify the susceptibility of mitochondria to mPT. During this experiment, mitochondria isolated from HEK293 cells and *A. franciscana* were exposed to Ca^2+^ pulses (Fig. S1a, b). Ca^2+^ accumulates in mitochondria until it triggers mPTP opening, followed by membrane depolarization, rupture of mitochondria, and release of sequestered Ca^2+^. Mitochondria isolated from HEK293 cells showed a jump in the fluorescence signal upon the addition of the fifth Ca^2+^ pulse that correlates with the mPTP opening (Fig. S1a). In contrast, mitochondria isolated from *A. franciscana* sequestered significant amounts of Ca^2+^ without a sudden increase in the fluorescence signal and mPTP activation (Fig. S1b), as reported earlier [40]. Additionally, the CsA treatment did not affect Ca^2+^ uptake in *A. franciscana* mitochondria, while it caused a delay in mPTP opening in HEK293 (Fig. S1a, b).

CsA inhibits mPTP in mammals by directly interacting with the mPTP activator, mitochondrial matrix protein CypD [50, 51]. CypD was shown to specifically interact with the mammalian ATP synthase peripheral stalk subunit OSCP [22] and induce ATP synthase leak channel activation [52, 53]. *A. franciscana* mitochondria were insensitive to CsA treatment despite the presence of CypD in their mitochondrial matrix [40]. However, the underlying molecular mechanism of the mitochondrial Ca^2+^ resistance and CsA-insensitivity in *A. franciscana* remains unknown. Thus, we performed an in-vitro structural and electrophysiology analysis of *A. franciscana* ATP synthase to find if it possesses any distinct properties that may contribute to mPTP inhibition.

### *A. franciscana* ATP synthase channel dwells in its inactive state and forms calcium-insensitive brief openings

*A. franciscana* ATP synthase was purified by solubilizing mitochondria with the non-ionic detergent n-dodecyl-ß-D-maltoside (DDM), pH 7.0. ATP synthase was purified by differential centrifugation and polyethylene glycol (PEG) precipitation followed by size exclusion chromatography (SEC) as described in Materials and Methods (Fig. S2a, b). The purified *A. franciscana* ATP synthase was fully assembled, as verified by the SEC profile (Fig. S2b). It demonstrated oligomycin-sensitive ATP hydrolysis activity, confirming the fully assembled and coupled conformation of purified ATP synthase (Fig. S2c). The planar lipid bilayer recordings revealed significant differences in *A. franciscana* and porcine heart ATP synthase leak channel activities. *A. franciscana* ATP synthase formed voltage-dependent brief openings with the channel mainly dwelling in its closed, inactive state (Fig. 1d–g). Figure 1h shows a continuous single-channel recording of *A. franciscana* ATP synthase before and after Ca^2+^ addition. The channel remained closed or had brief, infrequent openings in the presence of Ca^2+^, in contrast to what was observed for porcine heart ATP synthase under the same conditions [33] (Fig. 1i). The porcine heart ATP synthase was previously shown to form voltage-dependent channels with 1.5 nS peak conductance activity and prolonged dwell times in its open state in patch-clamp recordings [33]. In addition, Ca^2+^ was shown to increase the frequency of channel opening [33]. Figure 1i shows a continuous lipid bilayer recording of the porcine heart ATP synthase in the presence of 1 mM Ca^2+^, which forms large multi-conductance channels inhibited by ATP. BA was added to rule out the possibility of other mitochondrial ion channels, such as ANT, contributing to the recorded currents. BA failed to inhibit the channel, proving that channels are formed by porcine ATP synthase (Fig. 1i).

These results suggest that the *A. franciscana* ATP synthase leak channel has significantly different channel properties than the porcine heart ATP synthase. The former mainly dwells in its inactive form during the recording and is insensitive to Ca^2+^.

### The overall structure of *A. franciscana* ATP synthase at pH 7.0

**Fig. 2:**
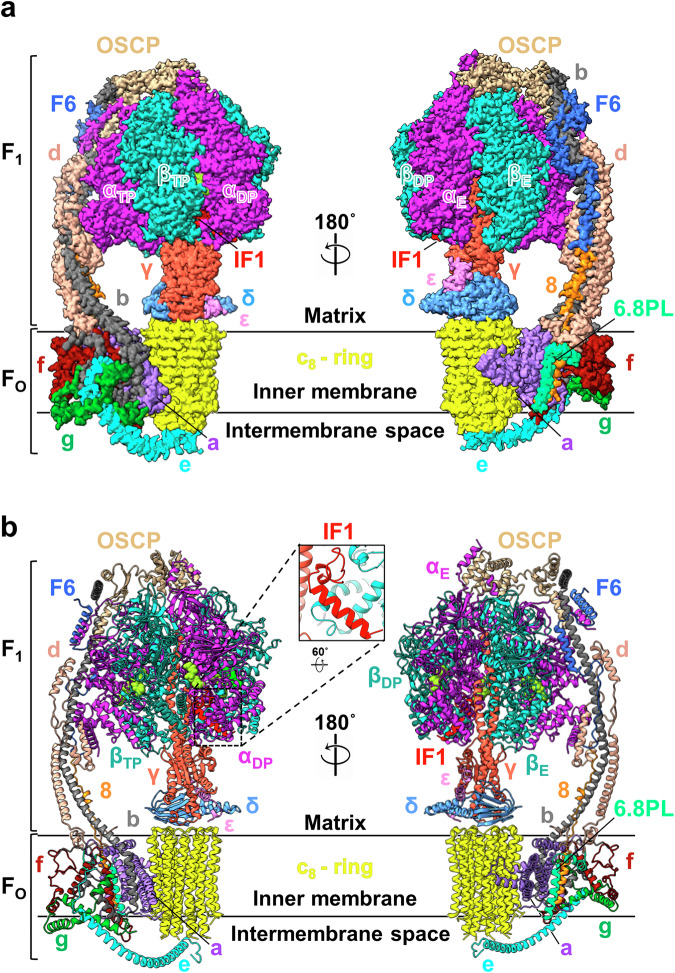
The overall structure of *A. franciscana* ATP synthase, purified at pH 7.0. **a** Side views of the cryo-EM composite map of *A. franciscana* ATP synthase monomer in rotational state 2 shown in surface representation. Monomeric ATP synthase contains 17 different subunits, shown in different colors. **b** Cartoon representation of the atomic model of *A. franciscana* ATP synthase monomer in rotational state 2 after fitting into the cryo-EM map shown in (**a**). ATP molecules occupying the nucleotide binding sites of β subunits are shown as lime green spheres. A close-up view of IF1 is shown in the black box.

We determined the complete structure of *A. franciscana* ATP synthase monomer using single-particle cryo-EM (Fig. 2) to study the structural basis of ATP synthase leak channel inactivation in this organism.

We obtained three cryo-EM maps for the *A. franciscana* ATP synthase, purified at pH 7.0 for the rotational states 1, 2, and 3a at nominal resolutions of 2.6 Å, 2.6 Å, and 2.7 Å, respectively (Figs. S3 and S4; Table S1). Each catalytic cycle of ATP synthase for ATP synthesis or hydrolysis is accompanied by the ~120° rotation of the central stalk γ subunit, allowing the formation of rotational states 1, 2, and 3 [44]. The rotational states differ from each other by the position of the rotor (consisting of γ, δ, ε subunits forming the central stalk and c_8_ ring) relative to the stator [44] (comprising of the α_3_β_3_ hexamer and peripheral stalk).

State 2 was the most prevalent in this dataset, which we selected for further analysis (Fig. S3). Local refinements of the F_1_ and F_O_ domains were performed to improve the map quality and resolution. The final local refined maps for  F_1_ and F_O_ at state 2 had nominal resolutions of 2.6 Å and 3.9 Å, respectively (Figs. S3 and S4; Table S1). The F_1_ and F_O_ local refined maps were combined to generate the composite map of *A. franciscana* ATP synthase (Fig. 2a). The local refined map for F_O_ at state 1 had a resolution of 3.3 Å (Table S1). *A. franciscana* maps had densities for seventeen ATP synthase subunits, reported in mammalian ATP synthases, except for subunit DAPIT, which most likely dissociated during the purification procedure, as noted in other cases [47].

The amino acid sequences used for model building for most ATP synthase subunits were obtained through annotation of the *A. franciscana* genome (Table S2).

The final structure of *A. franciscana* ATP synthase has a subunit composition similar to mammalian ATP synthases [46–48] (Fig. 2a, b). It consists of F_1_ and F_O_ domains connected by the central and peripheral stalk subunits. F_1_ consists of the α_3_β_3_ complex and central stalk subunits γ, δ and ε. The non-catalytic and catalytic nucleotide binding sites are located in α and β subunits, respectively. F_O_ consists of the c_8_-ring and subunits a (6 or ATP6), 8 (ATP8), e, f, g, and 6.8PL (6.8 kDa proteolipid). The peripheral stalk subunits are oligomycin sensitivity-conferring protein (OSCP), b, d, and F_6_. The 6.8PL-subunit sequence was not found in the *A. franciscana* genome annotation database. Thus, the poly-A chain was used to build the model of this subunit (Table S2). The sequence of the inhibitory factor 1 (IF1) subunit was also not found in the annotation database. The *Danio rerio* (zebrafish) model of IF1 (AlphaFold database) was used for fitting into the appropriate density in the *A. franciscana* ATP synthase map (Fig. 2b, Table S2).

ATP synthase is a unique molecular engine that uses the rotation of its subunits to perform the catalytic reaction of ATP synthesis and hydrolysis [44, 54, 55]. The proton translocation through F_O_, at the interface of subunits a and c, drives the rotation of the rotor subunits (c-ring and the central stalk) during the ATP synthesis reaction [55]. According to the “binding change mechanism [56],” the clockwise rotation of γ subunit within the α_3_β_3_ hexamer (viewed from the F_O_ side) drives the conformational changes in αβ pairs. Subsequent changes in β catalytic nucleotide-binding site affinities result in ATP synthesis [56, 57]. The β-subunits cycle through the conformational changes during ATP synthesis by adopting an open conformation (empty, β_E_ state), “loose” closed conformation (ADP-bound, β_DP_ state), and “tight” closed conformation (ATP bound, β_TP_) states. These events occur in the opposite direction (β_E_ → β_TP_ → β_DP_) during the ATP hydrolysis reaction upon counter-clockwise rotation of γ subunit within the α_3_β_3_ hexamer (viewed from the F_O_ side) [56, 57].

In the maps of *A. franciscana* ATP synthase (state 2), each non-catalytic nucleotide-binding site in α-subunits is occupied by an ATP molecule with an accompanying Mg^2+^ ion (Fig. 2a, b). They are named according to the β-subunits they interact with. The catalytic nucleotide-binding site in the β_TP_ subunit has ~50% occupancy with ATP and ~50% with ADP, based on the weak density of the gamma phosphate present on this site. This can be explained by the partial hydrolysis of endogenous ATP to ADP in the β_TP_ site during protein purification performed without added nucleotides. The nucleotide-binding site in the β_DP_ subunit is occupied with ADP, and the site on β_E_ is empty in all the maps (Fig. 2a, b). Therefore, the β_E_ subunit is found in an open conformation in all identified rotational states, while the β_TP_ and β_DP_ subunits are in the closed conformation. The significant structural differences between the β_TP_ and β_DP_ subunits, related to the “tight” and “loose” conformations found in mammalian ATP synthases [44], have not been observed in *A. franciscana* ATP synthase maps (Fig. S5a, b).

The intrinsic inhibitory factor IF1 is present in the *A. franciscana* ATP synthase maps for all three rotational states (state 1, state 2, and state 3a) in the pH 7.0 dataset. IF1 forms inactive monomers, active dimers, and inactive tetramers at different pH values in mammalian species [58, 59]. It forms active dimers at pH 7.0, where each IF1 monomer can interact with one ATP synthase molecule. The C-terminal regions of IF1 participate in dimer formation, while the N-terminal regions constitute the inhibitory part of the protein. The N-terminal region occupies the grove formed by the C-terminal domains of α- and β-subunits in bovine ATP synthase [59]. The N-terminal region of bovine IF1 was reported to be intrinsically disordered when it is not bound to F_1_. It folds in stages into the α-helical structure upon binding to ATP synthase, reaching its extensively folded state in the fully inhibited ATP synthase complex [57]. In *A. franciscana* ATP synthase, IF1 is localized at the interface between α_DP_ and β_DP_ subunits in all rotational states (Fig. 2, Fig. S5c–f). The N-terminal region of IF1 (residues 4-20) is unfolded in *A. franciscana* ATP synthase structure (Fig. 2 and S5c–f). At the same time, residues 27 to 45 form a fully folded α-helical C-terminal region and a partially folded segment (residues 21 to 26) located close to the γ subunit.

Interestingly, the IF1 dimer density was found in some F_1_ local refined maps of *A. franciscana* ATP synthase (Fig. S5f). Due to the low density of this region, it remains inconclusive whether the association of IF1 dimers to ATP synthase monomer is a specific feature of *A. franciscana*.

### The structure of *A. franciscana* ATP synthase peripheral stalk

The comparative structural analysis of mammalian and *A. franciscana* ATP synthases revealed differences in peripheral stalk conformation and its interaction with F_1_ and F_O_ domains (Fig. S6).

The *A. franciscana* and human ATP synthase maps (State 2) were aligned in relation to the central stalk for structural comparisons of the peripheral stalk and F_O_ domain (Fig. S6a). The peripheral stalk in human ATP synthase is shifted towards the central stalk (CS) compared to *A. franciscana*, which has a more relaxed conformation (Fig. S6a, Video S1). A similar shift is observed in the membrane-embedded F_O_ subunits in human ATP synthase (Fig. S6a, Video S1).

The comparison of *A. franciscana* and human subunits d and α_TP_ did not show an apparent structural difference when individual chains were aligned together (Fig. S6b). Subunits d and α_TP_ are shifted towards the central stalk in human compared to *A. franciscana* ATP synthase when the structures are aligned by the CS (Fig. S6c, d). The α-helical segment of the *A. franciscana* d-subunit (dA1-dL42) has a more upright conformation than the corresponding segment in the human d-subunit (dL5-dA43), which is tilted towards α_TP_ and CS (Fig. S6c, d).

In contrast to the human structure, α_TP_ in *A. franciscana* is moved towards the d-subunit, which shortens the distance between the α_TP_ and d chains (Figs. S6c, d and S7a, c). The shorter distance facilitates interactions between the N- and C-terminal domains of α_TP_ with the subunits d and b in *A. franciscana* (Fig. S7c, d). The N-terminal αE7 residue interacts with dT61 through a hydrogen bond and may form a salt bridge with bR166 (Fig. S7b). dT61 may also form a hydrogen bond with bE162 (Fig. S7b). This interaction is absent in human ATP synthase due to alanine residue (A61) at the dT61 position (Fig. S7d). A hydrogen bond may form between human dN59 and bQ162 (Fig. S7d).

The C-terminal regions of α_TP_ and d-subunits interact through a salt bridge between dR39 and αE467 in *A. franciscana* (Fig. S7b). This interaction is absent in human ATP synthase (Fig. S7d).

The comparison of *A. franciscana* and human b subunits shows a more relaxed conformation of the b-subunit segment consisting of residues P1-H145 in *A. franciscana* when ATP synthase structures are aligned by the CS (Fig. S6e). On the contrary, the corresponding segment in the human b-subunit (P3 to V147) has a more strained structure (Fig. S6e). This difference is less apparent when individual b chains are aligned (Fig. S6e).

Stronger interactions are observed between the subunits a and b in *A. franciscana*, compared with human ATP synthase. The α-helical segment of a-subunit in *A. franciscana* (a181-218, shown in blue in Fig. S8a) has a more linear structure compared with the corresponding segment in human ATP synthase (residues a185-225 shown in blue in Fig. S8b), which is more curved. A disulfide bond may form between the aM196 and bM66 in *A. franciscana*. Two hydrogen bonds may form between the bT59 and aQ191 in *A. franciscana* structure. Hydrophobic interactions occur between the a-subunit segment V188-M196 and bA62-M66 in *A. franciscana* (Fig. S8a). In human ATP synthase, the observed interaction is the salt bridge between the bD56 and aH172 (Fig. S8b). A hydrogen bond may form between bS62 and aT200. Additionally, π-π stacking interactions may form between the bF58 and aF193 in human ATP synthase. Stronger interactions between subunits a and b observed in *A. franciscana* may contribute to the more linear conformation of its b-subunit, as described above.

Two native cardiolipin molecules (CDL1 and CDL2) were found in the *A. franciscana* F_O_ domain between subunits a, b, f, and g (Fig. S8c). CDL1 is located between the subunits a, b, and f, interacting with the α-helical domains aH3, aH6, cH1, bH3, and fH4. The second cardiolipin molecule, CDL2, was found between the subunits b, f, and g and interacts with the helical domains fH1, fH3, fH4, bH1, bH2, and gH3 (Fig. S8c). Cardiolipin molecules were found at the dimer interface of ATP synthases in different species [48, 60], and have previously been reported to play a key role in stabilizing membrane protein oligomers [61]. However, CDL molecules were also found in the F_O_ domain of human ATP synthase monomer [47].

The observed differences in interactions between the peripheral stalk subunits b, d, and α_TP_ in F_1_, as well as subunits a and b in F_O,_ may contribute to a more rigid structure of the peripheral stalk in *A. franciscana* ATP synthase.

### The N-terminal helices of all α subunits have clear densities, suggesting stronger interactions with OSCP

**Fig. 3:**
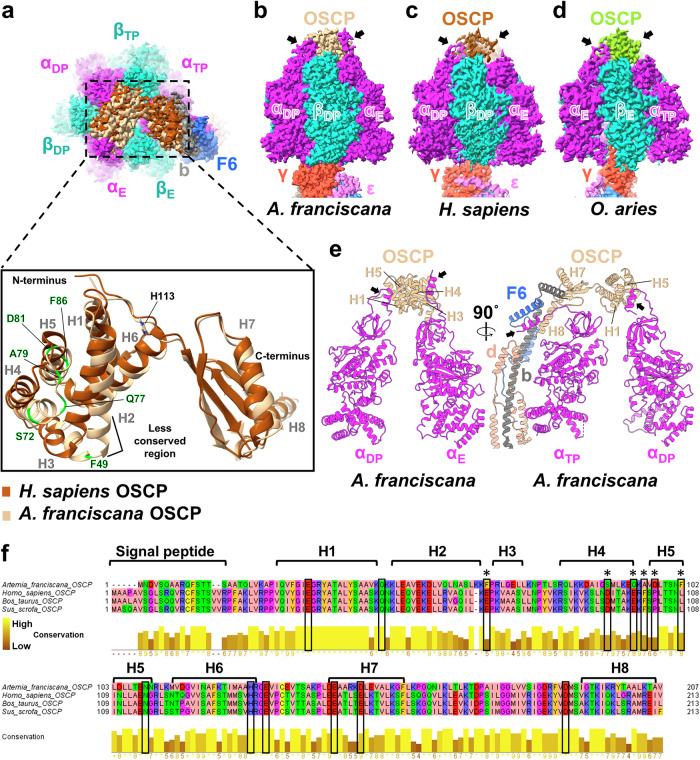
Distinct structural features of *A. franciscana* ATP synthase OSCP and α subunits. **a** The comparison of OSCP subunit structures in *A. franciscana* (PDB:9B0X, EMD-44061, state 2), and *H. sapiens* maps (PDB:8H9T, EMD-34581, state 2). The top views of superimposed maps and models of *A. franciscana* and *H. sapiens* F_1_ show the structural differences in OSCP subunits. OSCP has more open conformation in *H. sapiens* compared with *A. franciscana*. The side chain of the conserved OSCP H113 in *A. franciscana* is shown in the model. 6 non-conserved residues that may play a role in CypD binding to OSCP in mammals [22] are shown in green in *A. franciscana* model. Residue numbers exclude the signal peptide sequence. **b**–**d** Side views of the ATP synthase cryo-EM maps of *A. franciscana* (EMD-44061, state 2)*, H. sapiens* (EMD-34581, state 2) and *O. aries* (EMD-10573, state 1) showing differences in the interaction of α subunit N-terminal helical segments with OSCP. The N-terminal alpha-helical segments of all three α subunits interact with the OSCP only in *A. franciscana* ATP synthase. **e** Different side views of *A. franciscana* ATP synthase F_1_ domain showing interactions of α subunits with OSCP. For simplicity, β subunits and the central stalk subunits are not shown. **f** The amino acid sequence alignment of OSCP subunits of different species was generated through ClustalW Omega and treated using the Zappo color scheme in JalView, highlighting their physicochemical properties. Residue numbers include the signal peptide sequence. The 14 residues participating in the putative binding site of CypD with OSCP in mammals are shown in black boxes [22]. Six non-conserved residues in *A. franciscana* are indicated by asterisks. These residues are highlighted in green in (**a**).

Despite the overall structural similarities of the *A. franciscana* OSCP with its mammalian counterpart [46, 47], differences in OSCP interaction with α and peripheral stalk subunits were observed (Fig. 3). *A. franciscana* OSCP consists of N- and C-terminal domains, connected by the short linker (Fig. 3a). The N-terminal domain has six helices (H1-6) and interacts with the F_1_ α subunits. The C-terminal domain consists of a β-hairpin followed by two α helices, H7 and H8 (Fig. 3a). It is involved in the interaction with the peripheral stalk subunits F6, d, and b (Fig. 3a, b, e).

All three α-subunits in the *A. franciscana* ATP synthase maps have stronger densities for the first N-terminal α-helices. In contrast, the N-terminal α-helix in at least one of three α subunits was found to lack the clear density in the cryo-EM maps of most mammalian ATP synthases [46–49]. In the state 2 map of *H. sapiens* (human, EMD-34581) ATP synthase, the N-terminal densities were present for only α_TP_ and α_DP_ and absent for the α_E_ subunit [47] compared to the state 2 map of *A. franciscana* ATP synthase (Fig. 3b, c). All three N-terminal helices were omitted from the state 3a model of human ATP synthase (PDB:8H9U) due to weak or no densities found in α_TP,_ α_DP,_ and α_E_ subunits. Additionally, the density for the α_TP_ subunit was absent from the *Ovis aries* (ovine, EMD-10573) state 1 map (Fig. 3d) and human, bovine, and porcine ATP synthase maps (State 1 or 1a) [47–49].

Strong densities in cryo-EM maps usually correspond to more rigid regions within the protein. The presence of strong densities for all three α-subunit N-terminal helices in *A. franciscana* suggests that they are less flexible due to their enhanced interactions with the OSCP and other stator stalk subunits compared to mammalian ATP synthases.

In the state 2 model of *A. franciscana*, the N-terminal helix of α_DP_ closely interacts with the OSCP H1 and H5 helices (Fig. 3e). A salt bridge and a hydrogen bond may form between the α_DP_ E13 and OSCP H1 K28 residues and α_DP_ E7 and H1 Y18, respectively (Fig. S9a). Other salt bridges may form between the α_DP_ E7 and R95 located in the loop connecting H5 with H6 and between α_DP_ K15 and E92 in H5 (Fig. S9a). There are also hydrophobic interactions between α_DP_ and OSCP H1 and H5 helices that involve the following residues: αA10, L12, I16, L17, A19, and the OSCP L4 residue, OSCP H1 residues Y18, L22, A25 and H5 residues F86 and L89 (Fig. S9a).

The N-terminal helix of α_E_ interacts with the OSCP H3 and H4 helices (Fig. 3e). A salt bridge may form between the αE13 and OSCP R51 (Fig. S9a). Hydrophobic interactions involve the residues of α_E_ and OSCP H3 and H4 helices: αL12, I16, L17, A19, A20, and the H3 residues L52, L55. Other residues involved in hydrophobic interactions are H4 L65, A69, M73, and L61, located in the loop connecting the H3 and H4 helices (Fig. S9a). These interactions have not been found in human ATP synthase due to the lack of clear density for the α_E_ subunit N-terminal segment (state 2) [46, 47] (Fig. 3c).

The N-terminal helix of the α_TP_ subunit that is in down conformation interacts with the subunits d, b, F6, and C-terminal domain of OSCP (H8 helix) in *A. franciscana* (Fig. 3e). A hydrogen bond and a π-π stacking interaction may form between the OSCP H8 Y183 and F6 Q8 and the OSCP H8 Y183 and F6 F11, respectively (Fig. S9b). The distance between OSCP K125 and bE190 suggests a salt bridge formation in *A. franciscana* (Fig. S9b).

The interactions between the OSCP C-terminus and the α_TP_ N-terminal helix in *A. franciscana* are hydrophobic and involve the residues of the OSCP H7, H8 helices and connecting loop residues L135, F142, L163, V172, I180, A186, L187, A190, V191, M174, with the bM173, V174, I177, V178, I198, L194, L201, M204, as well as αA8, A10, V11, L12, I16, L17, L25 and F6 residues F11, L12, I15 (Fig. S9b).

The crucial role of the OSCP subunit in ATP synthase leak channel activation and mPTP formation has been shown recently [34, 62, 63]. The binding of CypD to the mammalian ATP synthase OSCP subunit activates the ATP synthase leak channel. In contrast, CsA, the binding partner of CypD, inhibits channel activation in mammals [22, 64]. However, the binding of CypD to OSCP in mammalian ATP synthase is only supported by immunoprecipitation, mutagenesis, and molecular docking studies [22, 46, 52, 63], with no structural information about the binding site in OSCP. Based on the ZDOCK server predictions, OSCP has to bend between its N- and C-terminal domains to accommodate CypD binding close to the conserved His112 [46] (His 113 in *A. franciscana*) (Fig. 3a). Moreover, protonation of His 112 was shown to mediate the mPTP inhibition [65].

*A. franciscana* mitochondria do not have CsA-sensitive and Ca^2+^-induced mPTP, despite the presence of CypD in the mitochondrial matrix [40]. The amino acid sequence and three-dimensional structural comparisons of the *A. franciscana* and human ATP synthase OSCP subunits revealed a less conserved H2 helix in *A. franciscana* (Fig. 3a, f). The H2 helix has more polar residues (Q, N, and S) and is shifted towards the β_E_ subunit in *A. franciscana* (Fig. 3a). Additionally, there is a slight shift in the H3 helix in *A. franciscana* structure towards the β_E_ compared to the human structure (Fig. 3a). A salt bridge may form between the OSCP H3 R51 and α_E_ E13 stabilizing the α_E_ in the up conformation in *A. franciscana* (Fig. S9a). This interaction and the overall density for the α_E_ N-terminal helix are absent in human ATP synthase [47].

Previous studies have reported the crucial role of the 14 conserved amino acid residues of OSCP in forming the putative binding site for CypD [22]. The amino acid sequence alignment of *A. franciscana* and mammalian OSCP subunits revealed that 6 of the 14 residues are not conserved in *A. franciscana* (Fig. 3a, f). These differences in the amino acid composition and structure of *A. franciscana* OSCP may interfere with CypD binding and explain the lack of sensitivity to CsA observed in this organism.

These observations allow us to conclude that the weaker interactions between the α subunit and peripheral stalk subunits found in mammalian ATP synthases could lead to higher flexibility of this region, initiating the transmission of conformational changes from F_1_ to F_O_ to activate the channel. In contrast, the strong N-terminal densities of all α-subunits and their enhanced interactions with the peripheral stalk subunits, OSCP, d, and F6 found in *A. franciscana*, may potentially prevent CypD or Ca^2+^-induced conformational changes in F_1_, and propagation of the signal through the peripheral stalk to F_O_ required for the ACLC activation.

### Distinct structural features of *A. franciscana* e-subunit may contribute to ACLC inactivation

The F_O_ domain of *A. franciscana* has the same subunit composition and overall structure as the mammalian ATP synthases. It consists of the c_8_-ring and a-subunit (subunit 6 or ATP6) that form the proton translocation pathway, as well as subunits 6.8PL, subunit 8 (ATP8), e, f, and g (Fig. 2a, b). The density of the DAPIT subunit was not found in our maps.

**Fig. 4:**
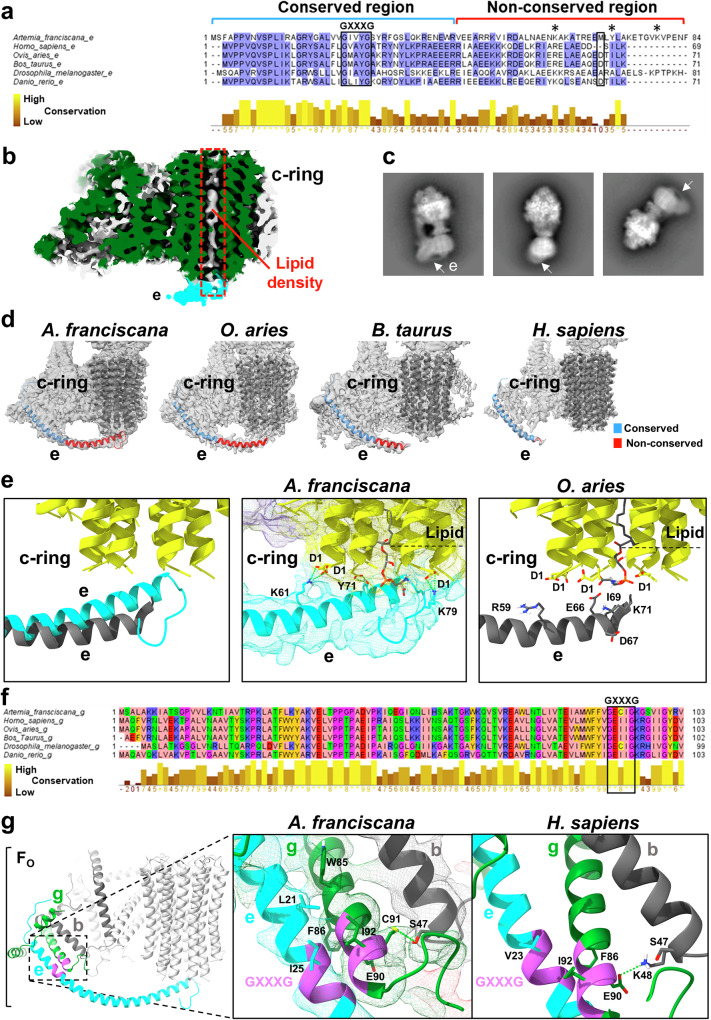
Distinct amino acid composition and structural features of *A. franciscana* ATP synthase e-subunit. **a** The amino acid sequence alignment of ATP synthase e-subunit in different species. *A. franciscana* has the longest sequence, containing 84 residues. The ClustalW Omega and JalView were used to treat the sequences. The regions with over 60% identity are highlighted in light blue using the BLOSUM62 score color scheme. The amino acid conservation level is labeled from brown (low conservation) to yellow (high conservation). The amino acid residues are conserved at the N-terminus of the e-subunit (residues 5-43, near subunit g), while residues at the C-terminus of the e-subunit (residues 44-84, near c-ring) are non-conserved. Residues of e-subunit participating in bond formation with the c-ring are shown with asterisks. Conserved GXXXG residues are shown in the black box. **b** The cryo-EM map of *A. franciscana* ATP synthase F_O_ domain (EMD-44061). The density corresponding to the lipids is colored grey. The C-terminus of the e-subunit that interacts with the c-ring and the lipids is colored cyan. **c** Different 2D classes of *A. franciscana* ATP synthase showing the density of e-subunit. **d** The comparison of F_O_ cryo-EM maps from different species are fitted with the respective models to show differences in the e-subunit C-terminus. The models of only e-subunit and c-ring are shown for simplicity. The C-terminal α-helical segment of the e-subunit is more extended and has a stronger density in *A. franciscana* (PDB:9BPG, EMD-44776, contour level 0.223), compared with the ovine (PDB:6TT7, EMD-10573, contour level 0.0251), bovine (PDB:6ZBb, EMD-11149, contour level 0.0108), and human (PDB:8H9F, EMD-34565, contour level 0.104) structures. The *A. franciscana* e-subunit is the longest and shows the closest interaction with the c-ring and the lipids occupying its cavity. The conserved (1-43) and non-conserved (44-84) residues in the e-subunit are shown in red and blue, respectively. **e** Left panel: The superimposed image of *A. franciscana* e-subunit (cyan) with the ovine e-subunit (grey) showing longer and curved conformation of the *A. franciscana* e-subunit and its closer positioning to the c-ring. Central and right panels: The possible interactions between subunit e, c-ring, and the lipid in *A. franciscana and O. aries*, respectively. The distances, shown by the green dotted lines, suggest multiple interactions in *A. franciscana* F_O_. Two salt bridges may form between residues eK61 and cD1 (*d* = 4.0 Å for both interactions). Two salt bridges may form between eK79 and D1 (*d* = 3.3 Å and *d* = 3.4 Å). Two hydrogen bonds may form between the eY71 and cD1 residues (*d* = 2.5 Å and *d* = 3.0 Å). The residues eK79 and eY71 may also interact with the lipids found inside the c-ring. The exact nature of the lipids occupying the *A. franciscana* c-ring remains to be identified. Lyso-phosphatidylserine was tentatively fitted into the density of the c-ring lumen at the intermembrane space side for visualization purposes only. These interactions cannot be formed between subunit e and c-ring residues based on the *O. aries* ATP synthase model. The terminal lysine residue in mammals was suggested to interact with the lipids occupying the c-ring [46]. **f** The amino acid sequence alignment of g-subunit in different species was generated through ClustalW Omega and treated using the Zappo color scheme in JalView, highlighting their physicochemical properties. The GXXXG motif shown in the black box has the conserved gI91 residue replaced by a cysteine in *A. franciscana* and *D. melanogaster*. **g** F_O_ domain of *A. franciscana* ATP synthase (PDB:9B0X, EMD-44061, contour level 11) showing the possible interactions between the GXXXG motifs of e- and g-subunits and g and b transmembrane helices. The gI91C substitution in *A. franciscana* favors hydrogen bond formation between the gC91 and bS47 (*d* = 3.4 Å). This interaction is not observed in the human ATP synthase (PDB: 8H9J) [47].

We found key differences in the *A. franciscana* e-subunit amino acid sequence while comparing it with its counterpart in human, ovine, bovine, zebrafish, and *Drosophila melanogaster*. The e-subunit sequence alignment revealed that the *A. franciscana* e-subunit has the most extended sequence among all these species, consisting of 84 amino acid residues. It is longer by 14 amino acid residues compared to mammalian e-subunits (Fig. 4a).

Furthermore, a stronger density of the e-subunit C-terminus was observed in the *A. franciscana* maps in contrast to mammalian structures (Fig. 4b-e). The density of the e-subunit was strong enough to be seen in the 2D classes of *A. franciscana* ATP synthase (Fig. 4c). The C-terminus was found to interact with the c-ring and lipids occupying its cavity, while the N-terminus interacts with subunits g, f, and b (Figs. 2b and 4b, d, e, g). Significantly weaker densities for the F_O_ e-subunit C-terminus were reported in the cryo-EM maps of mammalian ATP synthases, which are suggestive of higher flexibility in this region in mammals (Fig. 4d). In addition, the C-terminus of the *A. franciscana* e-subunit has a different conformation; it is more curved and extends towards the c-ring lumen compared with the ovine ATP synthase [46] (Fig. 4d, e).

The structural differences in the *A. franciscana* e-subunit could contribute to the enhanced interactions with the c-ring and lipids. The distances are suggestive of the following interactions between subunit e and the c-ring: Salt bridges between eK61 and cD1 residues, and eK79 and cD1, and a hydrogen bond between eY71 and cD1 may form in *A. franciscana* F_O_ (Fig. 4e). The residues eK79 and eY71 may also interact with the c-ring lipids (Fig. 4e, Video 1). The exact nature of the lipids occupying the *A. franciscana* c-ring remains to be identified. We have tentatively fitted phosphatidylserine (matrix side) and lyso-phosphatidylserine (intermembrane space side) into the densities of the c-ring lumen for visualization purposes only (Fig. 4e, Video 1). The lipids were omitted from the deposited models of *A. franciscana* ATP synthase. These lipids were provisionally modeled into the density of ovine ATP synthase c-ring previously [46]. Similar lipid densities were found to occupy the cavity of other mammalian ATP synthase c-rings [47, 48].

Stable interactions between subunits e and c may interfere with the rotation of the c-ring, enabling the e-subunit to act as an IF1 of F_O_ to stop the futile rotation of the c-ring. However, the aqueous environment of the intermembrane space may facilitate dynamic interactions between the e-subunit and c-ring, allowing the unimpeded rotation of the c-ring.

In the case of mammalian ATP synthases, the eY71 residue is substituted by isoleucine (I69 in ovine). In contrast, eK61 is substituted with arginine, another positively charged residue (R59 in ovine) [46, 47] (Fig. 4a); however, this interaction cannot form in mammals due to the larger distance between the subunits e and c. K79 in *A. franciscana* is not present in mammals since the mammalian e-subunits are shorter, consisting of 69-71 residues (Fig. 4a, e). The conserved C-terminal lysine residue is present in all mammalian e-subunits, which was suggested to interact with the aspartate residues at the bottom of the c-ring or with the lipids occupying it [46, 48]. In comparison with *A. franciscana*, weaker interactions between the e-subunit C-terminus and c-ring are present in mammals.

The e-subunit N-terminus interacts with the subunits g and b (Fig. 2). The important interaction site between the e- and g-subunits was reported to be the conserved GXXXG motifs present in both subunits [47] (Fig. 4a, f, g). These motifs form the compact triple transmembrane bundle with the b-subunit transmembrane helix, similar to mammalian ATP synthases [46]. In contrast to human ATP synthase structure [47], the C-terminus of the g-subunit in *A. franciscana* cryo-EM model is shifted up by one alpha-helical turn, which aligns the GXXXG motifs of e- and g-subunits together (Fig. 4g). This shift reduces the distance between the subunits and may favor the hydrophobic interactions (Fig. 4g). The amino acid residues before the GXXXG region in subunits e and g are hydrophobic, which can further stabilize the interactions between these two subunits (Fig. 4a, f, g). The GXXXG residues were selected to measure the distances between subunits e and g in *A. franciscana* and human ATP synthases (Fig. 4g). The distance between eG28 Cα and gG103 C is 4.5 Å in *A. franciscana* model (state 2, PDB:9B0X). In contrast, the distance between eG26 Cα and gG93 C is 7.8 Å in human ATP synthase (state 2, PDB:8H9J).

The C-terminal helical region of the b-subunit that interacts with the C-terminus of g is also shifted up by one helical turn in *A. franciscana* (Fig. 4g). The interaction between gE90 and bK48 in human ATP synthase is absent in *A. franciscana*. Meanwhile, another hydrogen bond may form between gC91 and bS47 in *A. franciscana*. This interaction is absent in human and ovine ATP synthases (Fig. 4g) since the Cys residue in the GXXXG region of the g-subunit in *A. franciscana* is substituted with the isoleucine in mammals (Fig. 4f, g). *D. melanogaster* is the only other species that has Cys at the g91 position (Fig. 4f). Interestingly, it also shares common properties of ATP synthase leak channel [42, 66] with *A. franciscana*, and is reported to lack the high-conductance channel activity of ACLC.

Overall, distinct structural features of the *A. franciscana* e-subunit and its enhanced interactions with the subunits c, g, b, and lipids within the c-ring lumen can contribute to the structural rigidity of the F_O_ domain and inhibition of the ATP synthase c-subunit channel in this organism.

### Proton translocation pathway in F_O_

The a-subunit (6 or ATP6) has a universal structure in mammalian species [46, 47, 67]. It is also well-conserved in *A. franciscana*, consisting of six horizontal α-helixes (α1-α6) that closely interact with the c-ring and define the proton translocation pathway within F_O_. The following conserved residues are involved in the proton translocation in mammalian ATP synthases [46–48] and are considered crucial for coupling ATP synthesis or hydrolysis with proton transport. The cE58 and aR159 residues serve as a “checkpoint” along with several negatively charged residues located within the a-subunit inlet (aE203) and outlet half-channels (aE145 and aD224). The latter residues participate in direct protonation-deprotonation of cE58 [46, 47](Fig. S10a, b). During ATP synthesis, the c-ring rotates clockwise within the hydrophobic lipid bilayer (viewed from the F_O_ side). The cE58 residue becomes protonated by accepting a proton from the aE203 when it reaches the hydrophilic inlet half-channel of the a-subunit [46, 47]. The inlet half-channel at the inner membrane starts with the aH168 and aH172 residues and ends with a203E [46, 67] in mammals (Fig. S10a). The mammalian residues cE58 and aH168 are conserved in *A. franciscana* (cE58 and aH166), while aH172 is replaced with threonine (aT170) (Figs. S10a and S11). A similar pattern is observed in *D. melanogaster* (Fig. S11). The c-ring rotation continues until protonated cE58 releases the proton into the matrix via the outlet half-channel of a-subunit [47] (Fig. S10b). The conserved aE145 and aD224 residues (aE143 and aE219 in *A. franciscana*) in the outlet half-channel are essential for accepting protons from cE58 before it encounters the positively charged residue aR159 [46] (aR157 in *A. franciscana*) (Fig. S10b). aR159 is located in the α5 helix of the human a-subunit and serves as a barrier to prevent uncoupling within the enzyme (Fig. S10b). The interaction between cE58 and aR159 is essential. It ensures that E58 is stripped of its proton before it leaves the outlet half-channel and can enter the subsequent protonation-deprotonation cycle. A salt bridge, however, does not form between the cE58-aR159 pair in mammalian ATP synthase [46] (Fig. S10b), in contrast to V-ATPases [68, 69], which were reported to have the second conserved barrier arginine residue in this region [70]. Similar to V-ATPases, the *A. franciscana* a-subunit also has two barrier arginine residues, R150 and R157, that separate the inlet and outlet half-channels of the a-subunit (Figs. S10b and S11a). A salt bridge may form between cE58 and aR157 in *A. franciscana* (Fig. S10b). The key direct proton donor/acceptor glutamates are also present in both half-channels in *A. franciscana* a-subunit (E198 in the inlet and E219 in the outlet) (Figs. S10 and S11a). These residues help to regulate the proton translocation in a certain rotational direction to ensure the coupling between the catalysis and proton translocation within the enzyme.

In ovine ATP synthase [46], water molecules were also proposed to be a proton acceptor in the outlet half-channel. The triad formed by Y221, S148, and Q152 was suggested to help coordinate the water molecules near the barrier aR159 [46]. The residues aS146, aY216, and R150 form this triad in *A. franciscana* (Fig. S10b). Based on the distance, a salt bridge may form between the residues aR150 and aY216.

### The overall structure of *A. franciscana* ATP synthase at pH 8.0

The binding of intrinsic inhibitory factor 1, IF1, to ATP synthase occurs in a pH-dependent manner in mammals [58, 59]. Thus, we determined the structure of *A. franciscana* ATP synthase at pH 8.0 to study if IF1 binding to ATP synthase is also pH-dependent in brine shrimp. *A. franciscana* mitochondria were solubilized with the non-ionic detergent DDM, followed by ATP synthase purification at pH 8.0.

For the pH 8.0 dataset, we obtained two 3D maps for the rotational states 1 and 2 with nominal resolutions of 2.53 Å and 2.87 Å, respectively (Figs. S12 and S13, Table S3). Further processing of maps for states 1 and 2 was performed to separate rotational states. We then obtained maps for states 1, 2, and 3a (Figs. S13 and S13), among which state 2 was the most prevalent under these conditions. The state 2 map was used for further analysis to generate the F_1_, F_O,_ and Peripheral Stalk (PS) local refined maps with nominal resolutions of 2.64 Å, 3.39 Å, and 4.01 Å, respectively. Local refinement was also performed for the F_1_, F_O,_ and PS for the other two rotational states 1 and 3a (Fig. S12).

The overall structure and subunit composition of *A. franciscana* ATP synthase at pH 8.0 (Fig. S14a, b) is similar to the one obtained at pH 7.0 (Fig. 2). The main difference  is the lack of IF1 in pH 8.0 structure in all rotation states, confirming the pH-dependent binding of IF1 to *A. franciscana* ATP synthase.

In the maps of *A. franciscana* ATP synthase (state 2, pH 8.0), each non-catalytic nucleotide-binding site in α-subunits is occupied by an ATP molecule with an accompanying Mg^2+^ ion (Fig. S5b). The catalytic nucleotide-binding site in the β_TP_ subunit has ~50% occupancy with ATP and ~50% with ADP, based on the weak density of the gamma phosphate present on this site, as was observed in the pH 7.0 structure (Fig. S5b). The nucleotide-binding sites in the β_DP_ and β_E_ subunits are empty in all the maps. Therefore, the β_DP_ and β_E_ subunits are found in an open conformation in all identified rotational states, while the β_TP_ subunit is in the closed conformation (Fig. S5b). The β_DP_ subunit is in the closed state in the presence of IF_1_ in the ATP synthase structure determined at pH 7.0. In contrast, it is in the open state in the absence of IF_1_ (pH 8.0 dataset, Fig. S5a–e).

We find that the overall structure of ATP synthase is more stable at pH 7.0 compared with 8.0. Although most ATP synthase particles show proper assembly at pH 8.0, one of the 3D classes (state 2, Class 3, 223,705 particles) does not have an apparent density for the peripheral stalk subunits b and d (Figs. S12 and S14c, d, Table S3). In addition to the peripheral stalk, the density for the subunits a, g, f, and 6.8PL is poorly defined, indicating higher flexibility and instability in these regions at pH 8.0 (Fig. S14c, d). Interestingly, a well-defined density for the e-subunit C-terminus is still present, even in the absence of apparent density for most of the peripheral stalk and F_O_ subunits (Fig. S14c, d). This further suggests the strong nature of interactions between the e-subunit and the c-ring, which may contribute to the inactivation of the ATP synthase leak channel in *A. franciscana*.

We also performed a comparative functional analysis of the single-channel activity of *A. franciscana* ATP synthase purified at different pH values. No significant differences were found in the peak conductance and open channel duration of *A. franciscana* ATP synthase in samples purified at pH 7.0 and 8.0 (Fig. S1c–h). Similarly, Ca^2+^ was still unable to induce channel activation in ATP synthase purified at pH 8.0 (Fig. S1e).

### ATP synthase e-subunit peptides inhibit the c-subunit channel activity

Human ATP synthase c-ring was shown to constitute the pore-forming unit of the ACLC. It forms voltage-gated large conductance channels with 1.5 nS peak conductance activity during patch-clamp and planar lipid bilayer recordings [32, 35].

**Fig. 5:**
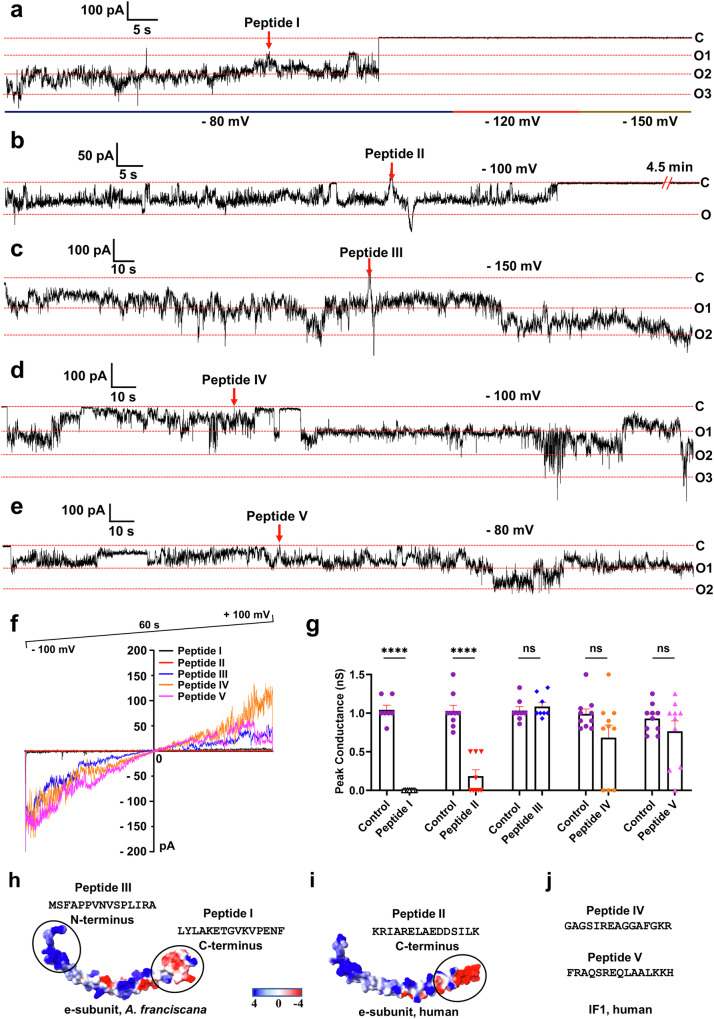
The C-terminal peptides *A. franciscana* and human ATP synthase e-subunits inhibit the human c-subunit channel activity. **a**–**e** Representative planar lipid bilayer recordings of c-subunit before and after adding 5 µM Peptides I, II, III, IV, or V. C refers to the closed and O to the open states of the channel. **f** Representative planar lipid bilayer recording of the c-subunit channel during continuous voltage ramp from -100 mV to +100 mV in the presence of Peptides I, II, III, IV or V. **g** Group data of the peak conductances of c-subunit channel activity before and after adding the Peptides I, II, III, IV and V (*n*  =  7 for Peptide I, *n*  =  9 for Peptide II, *n*  =  8 for Peptide III, *n*  =  10 for Peptide IV and *n*  =  10 for Peptide V; ****P  <  0.0001, paired *t*-test was used). **h**, **i** Electrostatic potential maps of *A. franciscana* and human e-subunits generated using APBS-PDB2PQR software. The models generated by AlphaFold were used to show the properties of the full-length protein structures. The C-terminus of *A. franciscana* e-subunit has both negatively and positively charged regions, while the C-terminus of the human e-subunit is more negatively charged. The shown sequences of e-subunits were used for peptide synthesis. **j** Peptides IV and V were designed by using the sequence of human IF1. Signals were filtered at 5 kHz using the amplifier circuitry.

We investigated if the peptide corresponding to the e-subunit C-terminal sequence would modulate the channel activity of the c-ring, given their strong interactions in *A. franciscana*. The c-subunit has an almost identical sequence and oligomeric state in *A. franciscana* and human ATP synthases [47] (Fig. 1, S11b). We, therefore, used purified human c-subunit (Fig. S2e-f and supplementary material) in our studies. Figure 5a shows a continuous lipid bilayer recording of the human c-subunit. The channel is activated upon voltage application and becomes fully inhibited upon the addition of the C-terminal peptide of *A. franciscana* e-subunit (Peptide I) in all recordings (*n* = 7) (Fig. 5a, f, g, h). We also tested the effect of human e-subunit C-terminal peptide (Peptide II) on the c-subunit channel activity. Peptide II fully inhibited the c-subunit channel in 5 recordings (Fig. 5b, f, g, i). The channel was partially inhibited in the other 4 recordings (Fig. S1i), confirming possible weak interactions between the e-subunit and the c-ring in mammals compared with *A. franciscana*. Notably, the human e-subunit is anchored to the membrane-embedded subunits of F_O_ and has a shorter length than the *A. franciscana* e-subunit, which may restrict its interaction with the c-ring in in vivo conditions. During planar lipid bilayer recordings, however, the peptide can freely diffuse and interact with the c-ring, leading to channel inhibition. Residues eR59 and eK69 may interact with cD1 in human ATP synthase. However, according to the electrostatic potential map of the human e-subunit, its C-terminus is more negatively charged, which may create repulsive forces between the atoms of the e-subunit and the c-ring (Fig. 5i). In contrast, the C-terminus of *A. franciscana* e-subunit has both negative and positive charges, which may strengthen the electrostatic complementarity and interactions between these two subunits (Fig. 5h).

The *A. franciscana* e-subunit N-terminal peptide (Peptide III), which does not have any interactions with the c-ring in our maps, was used as a negative control. Peptide III did not inhibit the c-subunit channel activity in any of the recordings (*n* = 8) (Fig. 5c, f, g, h), suggesting the requirement of specific interactions in channel inhibition. In addition, two other peptides with sequences corresponding to human IF1 were used as negative controls (Peptide IV and V, Fig. 5j). Peptide IV did not inhibit the c-subunit channel activity in 7 recordings, while it induced inhibition in 3 (Fig. 5d, f, g). Peptide V inhibited the channel in 1 recording, partially inhibited it in 2, and did not inhibit it in 7 recordings (Fig. 5e–g).

These results suggest that the interactions between e and c are essential for modulating channel activity. The weaker interactions between the human e-subunit and c-ring may become easily disrupted under pathological conditions inducing channel activation. On the contrary, stronger interactions observed in *A. franciscana* may prevent channel activation, suggesting the role of e-subunit as an inactivation gate of the ATP synthase c-subunit leak channel.

## Discussion

The exact molecular identity, composition, and structure of mPTP are still debated after several decades of extensive research. Recent studies suggested the plausible role of mitochondrial ATP synthase as a structural pore-forming unit of mPT [22, 23, 25, 26, 29, 32–35, 42, 46, 52, 71–73]. Nevertheless, the exact activation and inactivation mechanisms of the ATP synthase leak channel are not fully understood.

**Fig. 6:**
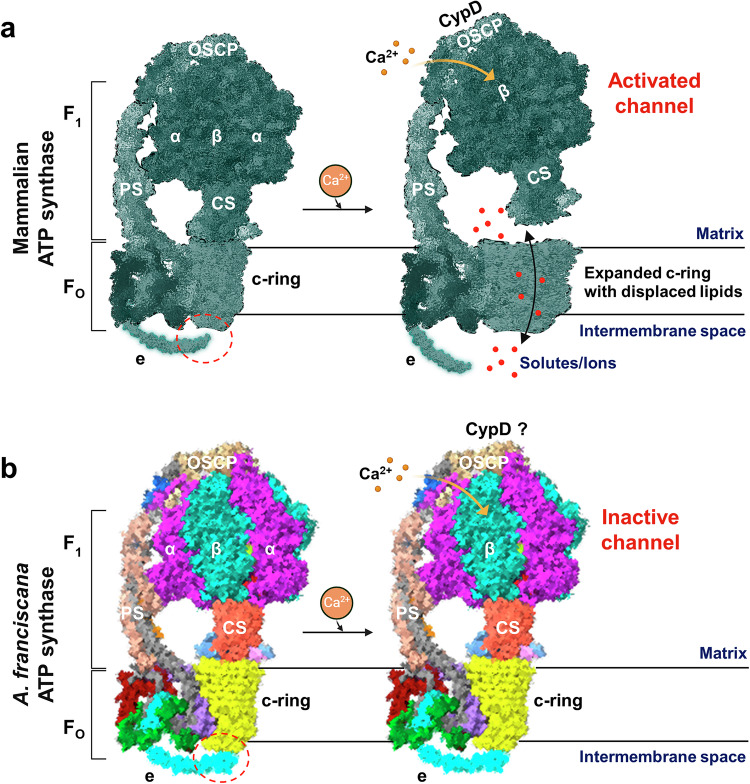
The hypothetical gating model of the ATP synthase c-subunit leak channel proposing the activation and inactivation mechanisms in mammalian and *A. franciscana* ATP synthase, respectively. **a** Hypothetical conformational changes in mammalian ATP synthase leading to activation of leak channel within its c-ring. The binding of Ca^2+^ to β-subunits and CypD to OSCP may induce conformational changes in F_1_ and peripheral stalk, inducing channel activation. Lipids in the c-ring lumen may be displaced or removed due to the expansion of the c-ring, allowing ion conduction. Black arrows indicate the possible path of ion flow through the channel. **b** Proposed leak channel inactivation mechanism in *A. franciscana*. Red dotted circles indicate the differences in interactions of e-subunit with c-ring in two species. Enhanced interactions between e-subunit with the c-ring and lipids may be key in regulating channel inactivation in *A. franciscana*. The figure is created with BioRender.com. For simplicity, only ATP synthase monomer is shown.

Different gating models of ATP synthase leak channel were proposed recently based on structural, electrophysiology, and cell death studies [8, 35, 42, 46, 74]. A large-conductance channel was suggested to form either by the ATP synthase c-ring [32, 33, 35, 75] or at the interface of two monomers [22, 23, 26]. The mPTP modulators, CypD and Ca^2+^, were shown to directly bind to ATP synthase subunits OSCP [22, 52, 65], and β [34], respectively, and induce conformational changes within the F_1_ domain. These changes may cause the detachment of F_1_ from F_O,_ activating the leak channel within the c-ring from the matrix side and highlighting the role of F_1_ as a gate of the ACLC (Fig. 6a). The signal may then propagate from F_1_ to the membrane-embedded F_O_ subunits e and g, through the peripheral stalk subunits OSCP, d, and b to activate the channel from the intermembrane space (Fig. 6a). Destabilization within the c-ring structure, due to detachment of F_1_ and changes in F_O,_ may occur, allowing widening of the pore followed by the channel activation [35, 75]. A “death finger” model was proposed earlier, highlighting the role of the e-subunit C-terminus in pulling the lipids out from the cavity of the c-ring [74]. The triple transmembrane helix bundle formed by the helices of subunits b, g, and N-terminal helix of e, known as the “hook apparatus,” was also suggested to facilitate lipid removal and channel activation [46].

The occlusion of the c-ring cavity with lipids has a crucial biological significance. The constitutively open leak channel within the c-ring would otherwise prevent generation of proton gradient across the mitochondrial inner membrane required for coupled respiration [56]. Changes in inner membrane permeability due to prolonged activation of mPTP are known to result in necrotic and apoptotic cell death in mammals [76]. In contrast, brine shrimp *A. franciscana* embryos resist anoxia-induced cell death and lack Ca^2+^-induced mPTP activation [40, 43]. Interestingly, the channel formed by *A. franciscana* ATP synthase dwelt predominantly in its inhibited state. It demonstrated transient, Ca^2+^-insensitive openings in planar lipid bilayer recordings, contrary to porcine heart ATP synthase. Ca^2+^, a key activator of the ATP synthase leak channel in mammals, binds to the βT163, known to coordinate Mg^2+^ ions during catalysis [34]. This residue is conserved in *A. franciscana* (βT160), suggesting that Ca^2+^ may bind to ATP synthase. Ca^2+^ binding, however, may not trigger conformational changes within ATP synthase necessary to activate the channel in *A. franciscana* (Fig. 6b). Enhanced interactions between the α and peripheral stalk subunits, OSCP, d, and F6 most likely do not allow the initiation and transmission of the signal from F_1_ to F_O_, preventing channel activation in *A. franciscana* (Fig. 6b).

In addition, the *A. franciscana* OSCP-subunit has a distinct amino acid sequence from that of mammals. 6 of the 14 conserved amino acid residues of OSCP, reported as being crucial in forming the putative binding site for CypD in mammals [22], are not conserved in *A. franciscana* (Fig. 3a, f). These differences may interfere with the binding of CypD to OSCP and explain the lack of CsA-sensitive mPTP in this organism (Fig. 6b).

*A. franciscana* ATP synthase e-subunit was found to be longer by 14 amino acid residues when compared with its mammalian counterparts. It consists of 84 residues and has a more curved C-terminal region that reaches towards the c-ring and the lipid plug (Fig. 4a–d). The C-terminal peptide of *A. franciscana* e-subunit inhibited the c-subunit channel activity in electrophysiology recordings, suggesting the role of e-subunit as a second gate of ACLC that acts in the intermembrane space. CRISPR-Cas9 genome editing studies of ATP synthase e-subunit in *A. franciscana* would provide further insights about the causative effect of e-subunit in contributing to the lack of Ca^2+^-sensitive mPTP in this organism. Additional studies are required to reveal whether other mitochondrial channels may play a role in Ca^2+^-resistant mPT in *A. franciscana*.

The ATP synthase e-subunit in *D. melanogaster* is also longer compared with mammals. It has 81 residues and shares similarities with *A. franciscana*, including a Cys residue in the g-subunit GXXXG motive (Fig. 4a, f). Purified ATP synthase from *D. melanogaster* forms small, only 53 pS channels [66] and lacks the large-conductance, 1.5 nS channel activity, further confirming the possible role of e-subunit in ACLC inactivation.

Furthermore, the e-subunit in *Trypanosoma brucei* ATP synthase is 92 amino acids long, with its C-terminus having a similar conformation to that of *A. franciscana*. It was reported to interact with the ten-stranded β-barrel structure of the c-ring [60]. The role of the ATP synthase leak channel and its contribution to mPTP still needs to be elucidated in this protozoan parasite.

The structure and conformation of the e-subunit may have evolved distinctly in different species to contribute to the physiological and pathological functions of the ATP synthase leak channel and its role in mPT.

mPTP plays a critical role in cardiomyocyte differentiation during embryogenesis [77]. Although mPTP opening is generally associated with cell death, a non-pathologic opening of mPTP was reported in the early embryonic heart [77]. Mouse heart cardiomyocytes at embryonic day (E) 9.5 displayed fragmented mitochondria with fewer cristae, depolarized mitochondrial membrane potential, higher levels of reactive oxygen species (ROS), and an open mPTP. Closure of mPTP at E11.5 lowered ROS and drove structural maturation of mitochondria, followed by cardiomyocyte differentiation [77]. Here, we can speculate that the shorter length of the e-subunit may enable physiological functions of mPTP during embryogenesis in mammals, meanwhile increasing the probability of channel activation and apoptosis under pathological conditions. In contrast, organisms like crustaceans and insects may have evolved to possess more rigid ATP synthase structures with a lower probability of channel activation due to the harsh environmental conditions they need to sustain. Perhaps a different channel assumed the physiological function of the mPTP in these organisms.

The link between the ATP synthase and mPTP channel has been recently questioned by a study that used genetic knockout of ATP synthase subunits g or F6 in HAP1-A12 cells [78]. Mitochondria isolated from HAP1-A12 Δg and ΔF6 cells were markedly sensitized to Ca^2+^-induced mPTP opening in CRC assay. In addition, the g-subunit was genetically depleted from the mouse cardiomyocytes [78]. Increased cardiomyocyte necrosis and larger infarcts were observed in Δg mice compared to controls [78]. These results allowed the authors to conclude that ATP synthase is a negative regulator of mPTP since its depletion sensitized mPTP opening [78]. It was assumed that the deletion of g and F6 subunits has entirely eliminated ATP synthase from the cell. Indeed, the levels of fully assembled ATP synthase oligomers, dimers, and monomers were significantly reduced upon deletion of g and F6 subunits. Yet, increased levels of the assembly intermediates containing the “free” c-ring were found in mitochondria [78]. The high levels of “free” c-ring, not assembled with F_1_, were reported to increase the probability of ACLC activation, predisposing neurons to cell death under glutamate excitotoxic conditions [35].

Furthermore, it has been reported that the deletion of any of the supernumerary membrane subunits of F_O,_ such as subunits e, g, DAPIT, and 6.8PL, is accompanied by the loss of the remaining supernumerary subunits [79]. This suggests that subunit g deletion in HAP1-A12 cells and engineered mice [78] would also reduce the levels of the other supernumerary subunits, including the e-subunit. Consequently, enhanced levels of “free” c-ring without either of its gates, F_1_ and e-subunit, would be present in mitochondrial inner membranes of HAP1-A12 cells and engineered mice. This would increase the probability of c-subunit leak channel activation, predisposing g-subunit depleted cardiomyocytes to cell death. Exacerbated necrotic damage was indeed observed in vivo in cardiomyocyte-specific Δg mice during the myocardial infarction induced by ischemia/reperfusion injury [78]. These findings support the current gating model of ACLC, suggesting that disassembly of ATP synthase dimers to monomers, followed by further disassembly of monomers to “free” c-ring, occurs during ischemic insults [35]. Aggravated necrotic damage reported in Δg mice also indirectly supports our proposed model, emphasizing the role of e-subunit in inactivating the ATP synthase leak channel in *A. franciscana*.

By possessing distinct structural features, *A. franciscana* ATP synthase achieves a higher level of structural rigidity, which enables this crustacean to withstand harsh environmental conditions that typically trigger the mPTP opening and pose challenges to mammalian organisms.

## Materials and methods

### Isolation of mitochondria from *A. franciscana*, HEK293 cells, and porcine heart

The dried eggs of *A. franciscana* were hydrated for 4 h in 0.25 M NaCl solution and then decanted and treated with Antiformin solution (1% Hypochlorite and 0.4 M NaOH) for 10 min. Eggs were then collected and washed with 1% sodium thiosulphate solution for 5 min. Further, washed eggs were incubated for ~14 h in 0.25 M NaCl solution in the shaker incubator before being harvested. The collected *A. franciscana* was resuspended in the Isolation Buffer A (500 mM sucrose, 150 mM KCl, 1 mM EGTA, 20 mM HEPES, 0.5% BSA, pH 7.5) and homogenized with pre-chilled Dounce homogenizer on ice. The homogenate was then centrifuged at 1000 *g* for 10 min at 4 °C, and the resulting supernatants were collected and centrifuged again at 1000 *g* for 10 min at 4 °C. The supernatant from the above step was then centrifuged at 9000 *g* for 20 min at 4°C. The resulting mitochondrial pellets were then resuspended in the Isolation Buffer B (500 mM sucrose; 150 mM KCl; 20 mM HEPES, 0.5% BSA, pH 7.5) and centrifuged at 9000 *g* for 20 min at 4 °C. The final mitochondrial pellets were then resuspended in 50-100 µl of Isolation Buffer C (500 mM sucrose; 150 mM KCl, 1 mM KH_2_PO_4_, 20 mM HEPES, 25 µM EGTA, pH 7.5) and used in different assays.

Mitochondria from HEK293 cells (obtained from ATCC) were isolated by using the Qproteome mitochondria isolation kit (Qiagen) following the manufacturer’s protocol.

Fresh porcine hearts were obtained immediately after slaughter, and the heart was finely minced in Buffer 1 (225 mM mannitol, 70 mM sucrose, 1 mM EGTA, and 20 mM Tris (pH 7.2)). The tissue was then homogenized with a Potter-Elvehjem tissue grinder, followed by centrifugation at 1000 *g* for 4 min. The supernatant was transferred into a fresh tube, and the sediment was homogenized for a second time to access intermyofibrillar mitochondria. After centrifugation at 1000 *g*, the supernatants were centrifuged at 9000 *g* for 10 min to sediment the mitochondria. The mitochondria were resuspended in Buffer 2 (225 mM mannitol, 70 mM sucrose, and 20 mM Tris (pH 7.2)) and centrifuged again at 9000 *g* for 10 min. The final mitochondrial pellet was suspended in 0.4 mL of Buffer 2. Isolated mitochondria were used immediately or stored at −80°C until further use.

### Calcium retention capacity assay

Calcium Retention Capacity (CRC) assay with HEK293 mitochondria (0.7 μg/μl) was performed in CRC buffer (150 mM sucrose; 1 mM KH_2_PO_4_, 1 mM MOPS, 5 mM succinate, 10 µM EGTA, pH 7.4) containing 20 µM rotenone (MP Biomedicals), and 1 µM Calcium Green-5N (Life Technologies). The following buffer was used for mitochondria isolated from *A. franciscana* (0.7 μg/μl) for CRC assay: 500 mM sucrose, 150 mM KCl, 1 mM KH_2_PO_4_, 20 mM HEPES, 5 mM succinate, 10 μM EGTA, pH 7.4, containing 20 µM rotenone and 1 µM Calcium Green-5N. 10 µM CsA (Sigma) was used when indicated. Data was acquired with a microplate reader (SpectraMax-iD3; Molecular Devices). 50 µM calcium pulses were added every 2 min while the fluorescence data were recorded at every 10 s interval. All the assays were done at 23 °C.

### Electron microscopy

Isolated mitochondria were fixed in a solution comprising 2.5% glutaraldehyde and 2% paraformaldehyde in 0.1 M phosphate buffer for 1 h (pH 7.4). The buffer-rinsed mitochondria were subsequently combined with 2% agarose and centrifuged. Following this, the chilled blocks were trimmed and further fixed in 1% osmium tetroxide for an additional 1 h. The samples underwent triple rinsing in 0.1 M phosphate rinse buffer. Subsequently, a graduated ethanol series and pure acetone were used for dehydration before embedding in LX-112 (Ladd Research, Williston, VT). Thin sections of a sample with 60 nm thickness were stained with uranyl acetate and lead citrate, then observed using a JEOL JEM1400 Transmission Electron Microscope (JEOL USA Inc., Peabody, MA, USA) at 60KV, located at the Penn State College of Medicine TEM Facility (RRID Number: SCR_021200). Images were captured using a NanoSprint43 Mk-II camera (Advanced Microscopy Techniques, Woburn, MA).

### Purification of human c-subunit, and *A. franciscana* and porcine heart ATP synthases

The human ORF ATP-synthase c1 (ATP5G1) subunit construct tagged on the C-terminus with Myc and DDK (Flag) was used (Origene Technologies). The construct for c-subunit was expressed in HEK293 cells and purified using the EZ view Red ANTI-FLAG M2 Affinity Gel (Sigma), according to the manufacturer’s protocol. Anti-c-subunit antibody (ab180149, Abcam) was used for protein detection.

Mitochondria isolated from porcine heart or *A. franciscana* were used for ATP synthase purification. Mitochondria were solubilized on ice by using DDM (1 g/g protein) for 1 h. Then, the suspension was centrifuged at 100,000 *g* for 1 h. Subsequently, 50% (w/w) PEG 6000 was added to the supernatant (final concentration, 7%). After 2 h incubation on ice, the protein precipitate was removed by centrifugation at 50,000 *g* for 1 h and PEG 6000 was subsequently added to the supernatant (final concentration, 2%). The protein precipitate was collected after incubation for 2 h on ice and centrifuged at 50,000 *g* for 1 h. The pellet, which contains ATP synthase, was solubilized in Buffer A (50 mM Tris-HCl, 100 mM NaCl, 2 mM MgSO4, 1 mM ATP, 0.05% DDM, pH 7.0 or pH 8.0). The solubilized sample was concentrated to 0.5 mL (Amicon centrifugal filters with 100 kDa molecular mass cutoff) and loaded onto a Superose 6 Increase 10/300 gel filtration column (GE Healthcare, USA) equilibrated with Buffer A. Fractions eluted at a retention volume of 11–12 mL were collected and used for further analysis.

### ATP hydrolysis activity measurements

ATPase activities were assayed in a buffer containing 50 mM Tris/H_2_SO_4_, 10 mM ATP, and 4 mM MgSO_4_, pH 8.0. The reaction was started by the addition of 5–10 μg ATP synthase solubilized in detergent and stopped after 10 min by the addition of SDS (final concentration, 10%, w/v). The released P_i_ was measured as described [80]. One unit of enzymatic activity (U) corresponds to 1 μmol of ATP hydrolyzed (equivalent to 1 μmol of P_i_ produced) per min, per mg protein. The effect of oligomycin on ATP hydrolysis activity was determined by adding the inhibitor to an ethanol solution (5 μg/mL, w/v).

### Peptide synthesis and validation

The peptides corresponding to the *A. franciscana* e-subunit C-terminal (LYLAKETGVKVPENF) and N-terminal (MSFAPPVNVSPLIRA) sequences, as well as human e-subunit C-terminal sequence (KRIARELAEDDSILK), were synthesized at the Penn State College of Medicine Macromolecular Synthesis Core. Peptides corresponding to the IF1 sequence GAGSIREAGGAFGKR and FRAQSREQLAALKKH were synthesized at LifeTein LLC. The peptide masses were validated by MALDI-MS.

### Planar lipid bilayer recordings

Planar lipid bilayer recordings were performed in intracellular solution (120 mM KCl, 8 mM NaCl, 0.5 mM EGTA, 10 mM HEPES, pH 7.3) by using α-L-phosphatidylcholine (Sigma) or α-L-phosphatidylcholine and cardiolipin (Avanti) in 3:1 ratio for forming the bilayer membrane. ePatch amplifier (Elements) was used for planar lipid bilayer recordings. Signals were filtered at 5 kHz using the amplifier circuitry. Purified ATP synthase or c-subunit were added on the *cis* side, and a constant voltage was applied to achieve protein insertion into the bilayer. ATP (1 mM), bongkrekic acid (BA, 10 µM), calcium (1 mM), and peptides I, II, III, IV and V (5 µM) were added on the *cis* side of the cuvette during the recordings without perfusion. To access the channel activity on both the voltage polarities, a voltage ramp was performed where the voltage was changed from -100 to +100 mV within 60 s. Clampfit software (Molecular Devices) was used for data analysis. The measured current was adjusted for the holding voltage, assuming a linear Current-Voltage relationship. The conductance (G) is expressed in pS, following equation *G* = *I/V*, where *I* is the peak membrane current in pA, and *V* is the membrane holding voltage in mV. Group data were quantified in terms of conductance and open channel duration. All population data were expressed as mean ± SEM.

### Sample preparation for single-particle cryo-EM

Grids were pretreated with Glow discharge under vacuum at 20 mA for 20 s. A 3uL aliquot of ATP synthase purified at pH 7.0 or 8.0 was applied onto a clean QuantiFoil R 2/1 Cu 300 mesh grid covered with 2 nm carbon. The grids were vitrified at 20 °C and 100% humidity using Mark IV Vitrobot (ThermoFisher). The excess solution was removed by 5 s of blotting before plunging into liquid ethane (−182 °C).

### Single-particle cryo-EM data collection and processing

CryoEM data for high-resolution analysis were acquired using a FEI Titan Krios G2 microscope (Thermo Fisher Scientific) operating at 300 kV, equipped with a Post-GIF K3 summit direct detection camera (TFS) and a Bio Quantum energy filter (20 eV slit width). The data collection process was automated by employing SerialEM. For the dataset at pH 7.0, a total of 12,547 movies were collected. These included 6,440 0° tilt movies and 6,106 20° tilt movies, each composed of 56 fractions resulting in a cumulative exposure time of 2.8 s, with 0.05 s per fraction. For the pH 8.0 dataset, a total of 12,097 movies were obtained. These included 5633 0° tilt movies and 6,464 20° tilt movies, each composed of 60 fractions resulting in a cumulative exposure time of 3.6 s, with 0.06 s per fraction. The nominal magnification was ×81,000, corresponding to a calibrated pixel size of 1.068 Å. The specimen was exposed at a rate of 15.8 electrons per pixel per second, with a total exposure of approximately 50 electrons per square angstrom. The defocus ranged from -1 to -1.7 μm for 0° tilt movies and from -0.7 to -1.5 μm for 20° tilt movies. The nominal magnification was ×81,000, corresponding to a calibrated pixel size of 1.068 Å. The specimen was exposed at a rate of 20.9 electrons per pixel per second, with a total exposure of approximately 50 electrons per square angstrom. The defocus ranged from -1 to -1.7 μm for 0° tilt movies and from −0.7 to −1.3 μm for 20° tilt movies. Details for each dataset are described in Tables S1 and S3.

All image processing procedures were executed using cryoSPARC, unless otherwise specified. Movie fractions were aligned utilizing MotionCor2 with a 5 × 5 grid, and parameters for the contrast transfer function (CTF) were assessed through patch CTF estimation in cryoSPARC. Particle selection was conducted employing a template-based approach, utilizing a previously resolved 4.3 Å porcine ATP synthase map to generate 50 2D projection images. The diameter of the particle was set to 180 Å and these were extracted using a box size of 400 pixels. Contamination and outliers were initially removed by running 3 rounds of 2D classification. “Clean” particles were used to generate an initial 3D map using the Ab initio algorithm.

For the pH 7.0 dataset, a total of 5.5 million particles were initially selected and subjected to a round of 2D classification using 4x binned particles, resulting in 2.7 million particles retained for subsequent 3D processing. These 2,683,006 particles were subsequently re-extracted with a voxel size of 512. A subset of 50,000 particles was utilized for reference-free ab initio reconstruction to serve as the reference map for subsequent heterogeneous refinement. Heterogeneous refinement led to the separation of three major rotational states, comprising 864,418 state 1 particles, 847,418 state 3 particles, and 970,828 state 2 particles. For each state, NU-refinement was employed, involving per-particle CTF refinement, spherical aberration fitting, tetrafoil fitting, and anisotropic magnification fitting, resulting in global structures with resolutions of 2.6 Å for state 1, 2.7 Å for state 3, and 2.6 Å for state 2. To obtain high-resolution F_O_ maps for state 1, particle subtraction was performed using an F_O_ mask to subtract the F_1_ signal from the particle stack, preserving only the F_O_ information. A subsequent round of heterogeneous refinement was executed, resulting in the selection of 673,185 F_O_ particles. Local refinement yielded a 3.3 Å state1 F_O_ map suitable for atomic model building. For state 3 and state 2 F_O_ maps, focused 3D classification processes with F_O_ mask were employed instead of particle subtraction. This process selected 637,185 state 3 particles and 722,737 state 2 particles, followed by F_O_ local refinement, resulting in 3.5 Å state 1 and state 2 F_O_ maps. The 3D classification was conducted for the particle stacks of each rotational state to further segregate sub-states or identify IF1 bound/unbound conformations. For state 1, four classes with 237,890 particles, 229,181 particles, 217,964 particles, and 179,725 particles were generated through the 3D classification process. Subsequent F_O_ or F_1_ local refinement provided F_O_ maps with resolutions ranging from 3.4 Å to 4.8 Å and F_1_ maps with resolutions between 2.6 Å and 3.6 Å. For state 3, a similar approach resulted in four classes with 252,379 particles, 233,700 particles, 187,161 particles, and 174,178 particles from the 3D classification. F_O_ or F_1_ local refinement produced F_O_ maps with resolutions between 3.8 Å and 4.9 Å and F1 maps with resolutions between 2.5 Å and 4.2 Å. For state 2, four classes with 290,002 particles, 232,736 particles, 225,659 particles, and 222,431 particles were generated through the 3D classification process. Subsequent F_O_ or F_1_ local refinement yielded F_O_ maps with resolutions ranging from 3.9 Å to 4.5 Å, and F_1_ maps with resolutions between 2.6 Å and 3.2 Å.

For the pH 8.0 dataset, a total of 5.5 million particles were initially selected and underwent a round of 2D classification with 4x binning, yielding 3.4 million particles for subsequent 3D processing. Among these, 3,381,825 particles were re-extracted with a voxel size of 512, and particles with high-resolution information were identified through two rounds of heterogeneous refinement. From the heterogeneous refinement, two major classes were distinguished, comprising 1,374,794 particles and 919,437 particles, which were further subjected to NU-refinement. This refinement involved per-particle CTF refinement, spherical aberration fitting, tetrafoil fitting, and anisotropic magnification fitting, resulting in structures with resolutions of 2.5 Å and 2.8 Å, respectively. Additionally, 3D classification was employed to segregate sub-states within both classes. For class 1, sub-classification led to the isolation of five different state 2 sub-states and one state 1 sub-state. These sub-states contained 302,409, 275,464, 223,705, 223,680, 214,385, and 135,151 particles, respectively. These were subsequently subjected to NU-refinement, yielding structures at global resolutions of 2.7 Å, 2.8 Å, 2.8 Å, 3.0 Å, 3.8 Å, and 3.0 Å. To generate high-quality maps for F_O_, F_1_, and the Peripheral stalk (PS), local refinements were performed with masked regions, resulting in maps at resolutions ranging from 3.3 Å to 4.4 Å for Fo, 2.6 Å to 3.8 Å for F_1_, and 3.8 Å to 4.9 Å for PS. For class 2, sub-classification revealed three different state 3 sub-states and one state 1 sub-state, comprising 284,505, 205,595, 195,711, and 233,566 particles, respectively. These were subsequently subjected to NU-refinement, resulting in structures at global resolutions of 2.8 Å, 3.8 Å, 3.7 Å, and 2.8 Å. Local refinements produced maps with resolutions ranging from 3.4 Å to 4.3 Å for F_O_, 2.7 Å to 4.0 Å for F_1_, and 3.9 Å to 4.6 Å for PS.

Details of the image process workflow are described in Figs. S3, S4, S12, and S13.

The sharpened maps obtained from F_1_ and F_O_ or F_1_, F_O,_ and PS local refinements were combined to generate composite maps in Phenix software.

### Annotation of *A. franciscana* ATP synthase genes

The amino acid sequences for *A. franciscana* ATP synthase subunits were obtained through the annotation of ATP synthase genes by analyzing the previously obtained and assembled genome of *A. franciscana* [81]. To retrieve the target genes, we first screened the predicted *A. franciscana* proteome using Blastp with known representative genes. The collected hits were then aligned and compared to the known genes described in other arthropods. The predicted *A. franciscana* genes that aligned well were used for further analysis. A tblastn was run by using the same representative target proteins directly onto the assembled genomic scaffolds to overcome the possibility that a gene would have been missed by the gene prediction. The hits were inspected to complete the gene models (extending to start/stop codon, joining exons in the case hits were reported in several pieces ( = exons)). The final annotated sequences with the highest identity percentage, alignment score, and query cover percentage were used for the atomic model building.

### Model building and atomic model fitting

The model building of *A. franciscana* ATP synthase subunits was performed by using annotated sequences as an input for the prediction in ColabFold [82] or Alphafold [83] software. The predicted models were processed to remove the signal peptides by comparing the amino acid sequence from *A. franciscana* with the sequences of ATP synthase from human, ovine, and bovine. The models were fitted into the cryo-EM map density through multiple rounds of rigid and flexible fitting in Coot and Phenix using real-space refinement and manual adjustments. The validity of the annotated sequences was confirmed by comparing the residues with the cryo-EM map density after fitting. We optimized the structure in the regions with poor density, comparing our map with maps and models from other ATP synthases. These structures were used as a reference during flexible fitting steps. The structures were validated using MolProbity, which mainly considered the Ramachandran plot, clash score, rotamers, and side chain outliers.

## Supplementary information

Video 1

Supplementary material

Supplementary information

Video S1

Video legend

## Data Availability

All cryo-EM maps and atomic coordinates, including entire and locally refined maps of ATP synthase, have been deposited in the Electron Microscopy Data Bank (EMDB) and the Protein Data Bank (PDB) under specific accession codes. pH 7.0 dataset: EMD-44087 (state 1, F_O_F_1_), EMD-49580 (state 2, F_O_F_1_, consensus map), EMD-44061/PDB:9B0X (state 2, F_O_F_1_, composite structure), EMD-44094 (state 3a, F_O_F_1_), EMD-44096 (state 3a, local-refined F_1_), EMD-44162 (state 2, local-refined F_O_), EMD-44165 (state 2, local-refined F_1_) and EMD-44776/PDB:9BPG (state 1, local-refined F_O_). pH 8.0 dataset: EMD-44173 (state 1, F_O_F_1_), EMD-49579 (state 2, F_O_F_1_, consensus map), EMD-44142/PDB:9B3J (state 2, F_O_F_1_, composite structure), EMD-43079 (state 3a, F_O_F_1_), EMD-44177 (state 2, F_O_F_1_ with a weak density of the peripheral stalk), EMD-44169 (state 2, local-refined F_O_), EMD-44170 (state 2, local-refined F_1_), EMD-44172 (state 2, local-refined peripheral stalk).
