# Supplementary figures and images for: Cryo-EM structure of the brine shrimp mitochondrial ATP synthase suggests an inactivation mechanism for the ATP synthase leak channel
Source: Cell Death Differ. 2025 Mar 19;32(8):1518–35. doi: 10.1038/s41418-025-01476-w (PMC12325954; doi:10.1038/s41418-025-01476-w)

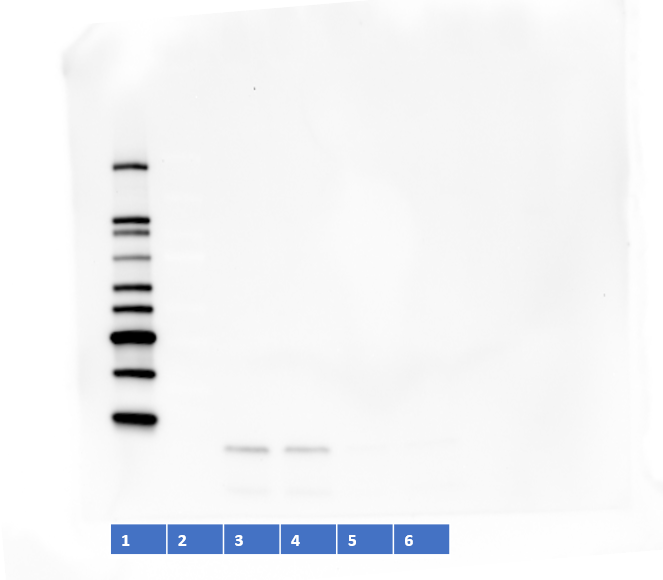


**50**

**Empty**

**20**

**100**

**220**

**Figure S2f**

**Purified c-ring**

**Purified c-ring**

**Empty**

**Protein Marker**

**kDa**

**60**

**80**

**120**

**40**

**30**

Supplement: Supplementary file 2 — Supplementary material [file 41418_2025_1476_MOESM2_ESM.docx]
