# Supplementary material for: Cryo-EM structure of the brine shrimp mitochondrial ATP synthase suggests an inactivation mechanism for the ATP synthase leak channel
Source: Cell Death Differ. 2025 Mar 19;32(8):1518–35. doi: 10.1038/s41418-025-01476-w (PMC12325954; doi:10.1038/s41418-025-01476-w)
Supplement: Supplementary file 3 — Supplementary information [file 41418_2025_1476_MOESM3_ESM.docx]

**Supplementary Information**

Cryo-EM structure of the brine shrimp mitochondrial ATP synthase suggests an inactivation mechanism for the ATP synthase leak channel

Amrendra Kumar^1#^, Juliana da Fonseca Rezende e Mello^1#^, Yangyu Wu^2#^, Daniel Morris^1^, Ikram Mezghani^1^, Erin Smith^1^, Stephane Rombauts^3,4^, Peter Bossier^5^, Juno Krahn^6^, Fred J. Sigworth^2^, Nelli Mnatsakanyan^1*^

^1^Department of Cellular and Molecular Physiology, Penn State College of Medicine, Hershey, PA, USA

^2^Department of Cellular and Molecular Physiology, Yale University School of Medicine, New Haven, CT, USA

^3^Department of Plant Biotechnology and Bioinformatics, Ghent University, Ghent, Belgium

^4^VIB Center for Plant Systems Biology, VIB, Ghent, Belgium

^5^Faculty of Bioscience Engineering, Ghent University, Ghent, Belgium

^6^ National Institute of Environmental Health Sciences, Durham, NC, USA

#These authors contributed equally

*Correspondence: [nmnatsakanyan@pennstatehealth.psu.edu](mailto:nmnatsakanyan@pennstatehealth.psu.edu)


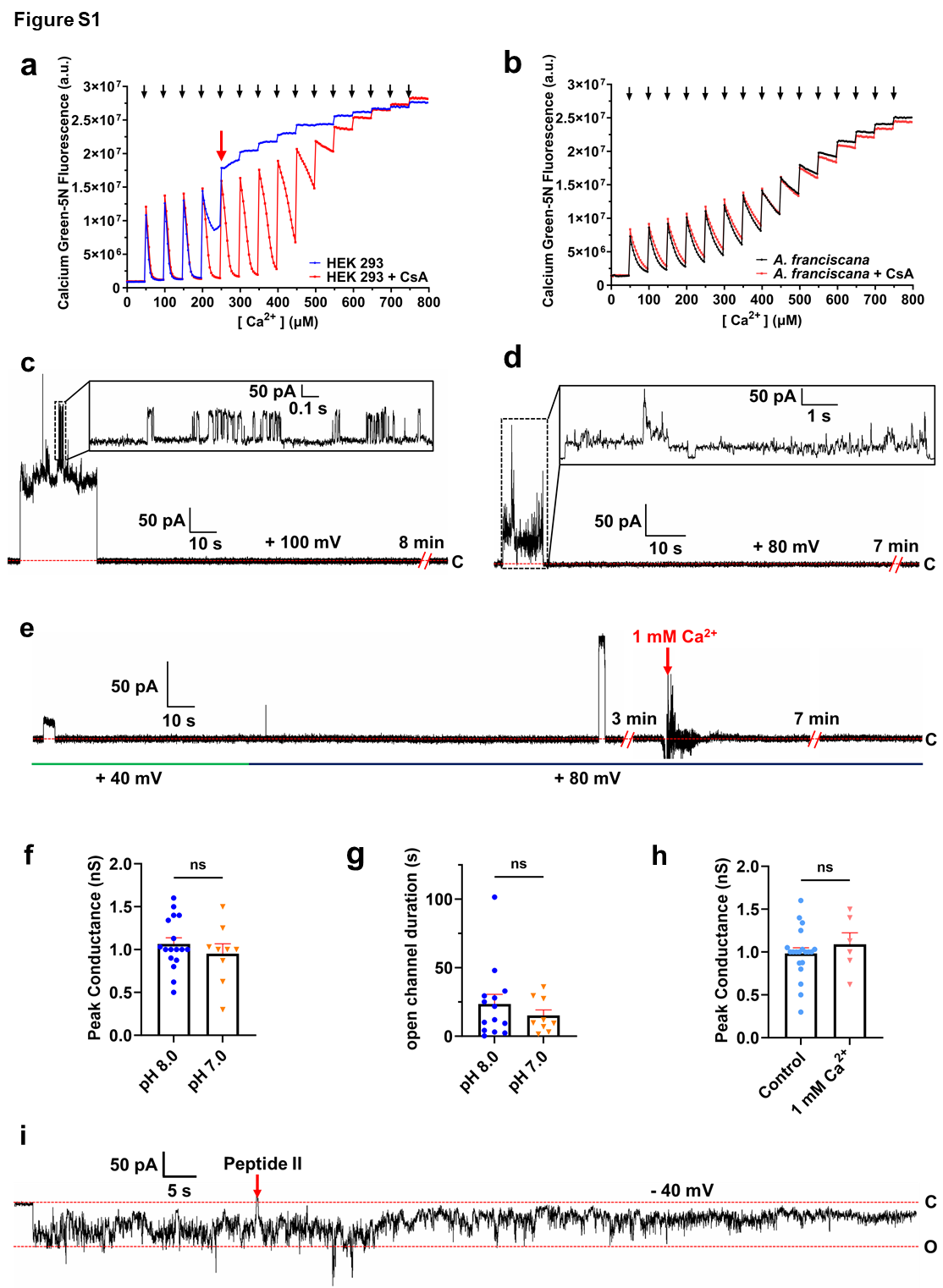


**Figure S1.** ***A. franciscana* mitochondria lack the Ca^2+^-induced and CsA-sensitive mitochondrial permeability transition and Ca^2+^-sensitive large conductance channel of ATP synthase. a,** Calcium retention capacity (CRC) assay of control and CsA-treated mitochondria isolated from HEK 293 cells and **b,** *A. franciscana*. The black arrows indicate the addition of 50 µM Ca^2+^ pulses. The red arrow indicates the change in fluorescence signal due to the mPTP opening in HEK 293, *n* = 3 biologically independent samples. **c,** Representative channel recordings of *A. franciscana* ATP synthase purified at pH 8.0. **d,** Representative channel recording of *A. franciscana* ATP synthase in the presence of 1 mM Ca^2+^. **e,** Representative channel recording of *A. franciscana* ATP synthase purified at pH 8.0 before and after adding 1 mM Ca^2+^. **f,** Group data of peak conductance activities and **g,** open channel duration of *A. franciscana* ATP synthase purified at pH 8.0 and pH 7.0. **h,** Group data of peak conductance activities of *A. franciscana* ATP synthase channel (purified at pH 8.0) in the presence and absence of 1 mM Ca^2+^. An unpaired *t*-test was used for statistical analysis. **i,** Representative planar lipid bilayer recordings of human c-subunit before and after adding Peptide II (5 µM). Signals were filtered at 5 kHz using the amplifier circuitry.


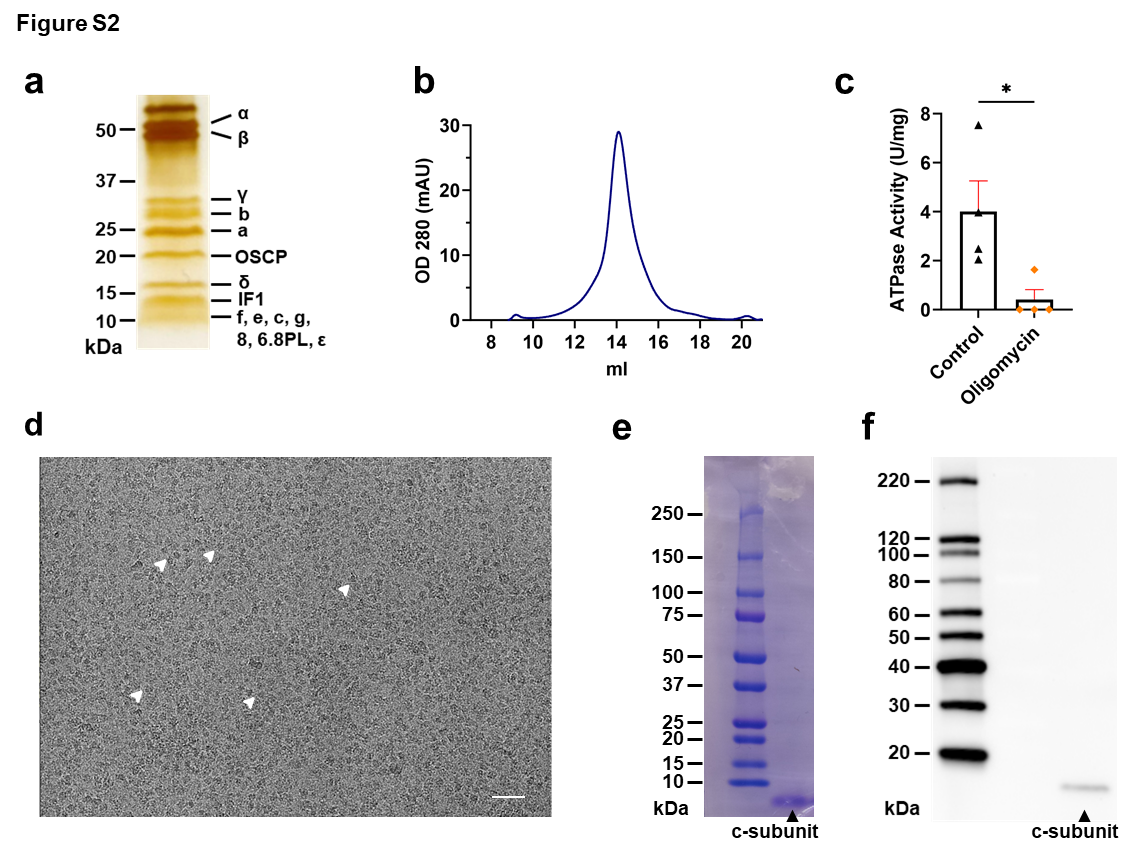


**Figure S2.** **Purification of *A. franciscana* ATP synthase and human c-subunit. a,** SDS-PAGE analysis of DDM-purified *A. franciscana* ATP synthase. The silver stain was used for protein visualization. **b,** Size exclusion chromatography (SEC) profile of purified *A. franciscana* ATP synthase monomer. A representative example of three independent runs is shown. **c,** Oligomycin-sensitive ATP hydrolysis activity was measured to assess the level of coupling of purified *A. franciscana* ATP synthase. The assay was performed at 37ºC in the absence and presence of oligomycin (*n* = 4, **P =* 0.033). **d**, Representative cryo-EM micrograph showing the homogenous distribution of *A. franciscana* ATP synthase particles on the grid. ATP synthase particles are shown by the white arrowhead. Scale bar, 20 nm. **e**, SDS-PAGE analysis of purified c-subunit from HEK293 cells. The protein marker (Precision Plus) and purified c-subunit band are visualized by Coomassie-Blue staining. **f**, The immunoblot analysis of purified c-subunit form HEK293 cells. The membrane was probed with an anti-c-subunit antibody. The protein marker MagicMark XP is shown on the first lane.


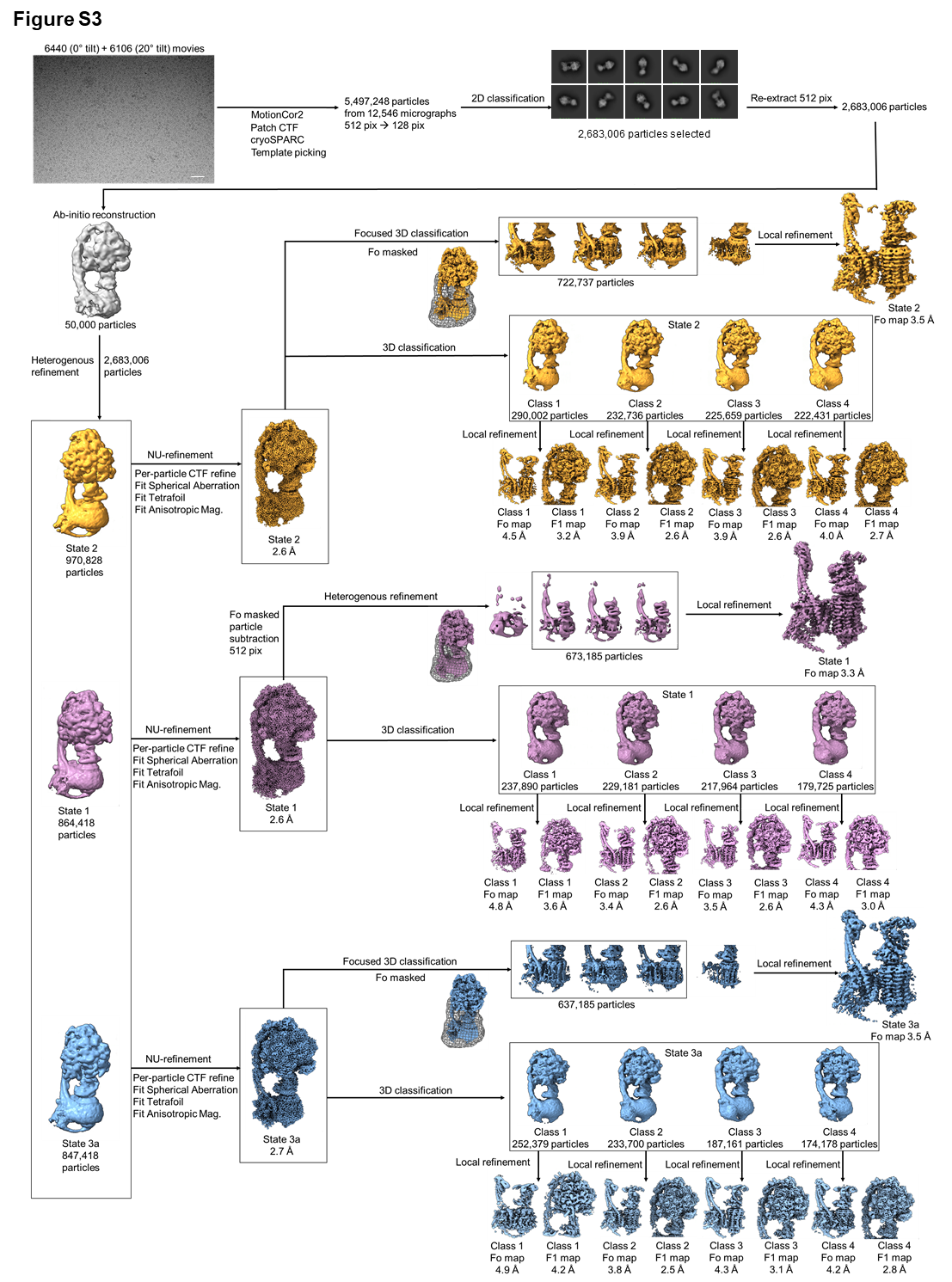


**Figure S3. Cryo-EM data processing of *A. franciscana* ATP synthase purified at pH 7.0.** The data processing flowchart of *A. franciscana* ATP synthase in three rotational states, state 1, state 2, and state 3a. The local refinement of the local F_O_ and F_1_ regions in three rational states was performed. Scale bar, 20 nm.


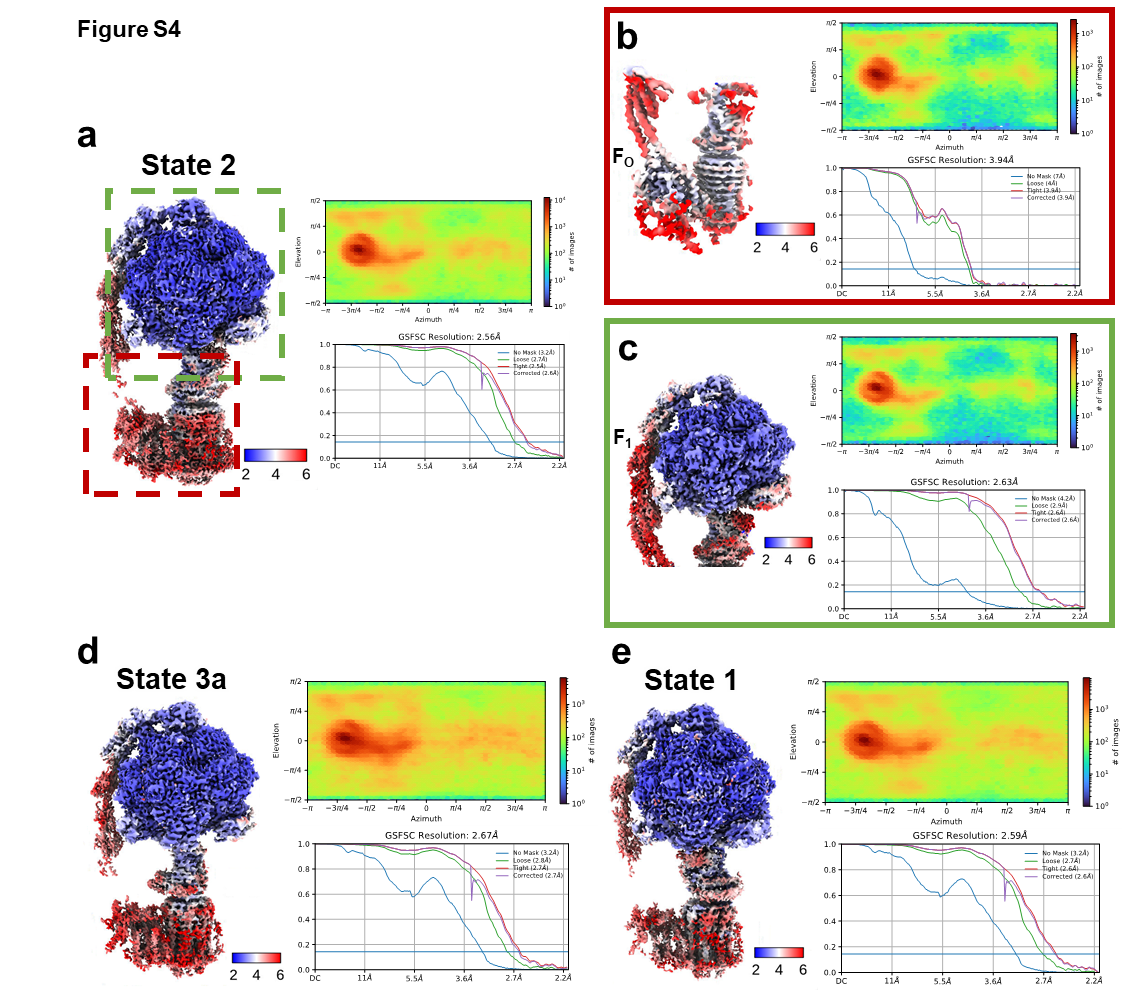


**Figure S4.** **Local resolution estimation, particle distribution, and Gold Standard Fourier Shell Correlation (GSFSC) resolution estimation of the *A. franciscana* ATP synthase (pH 7.0.) in different rotational states**. **a**, State 2, **b**, State 2 F_O_ local-refined, **c**, State 2 F_1_ local-refined, **d**, State 3a, **e**, State 1.


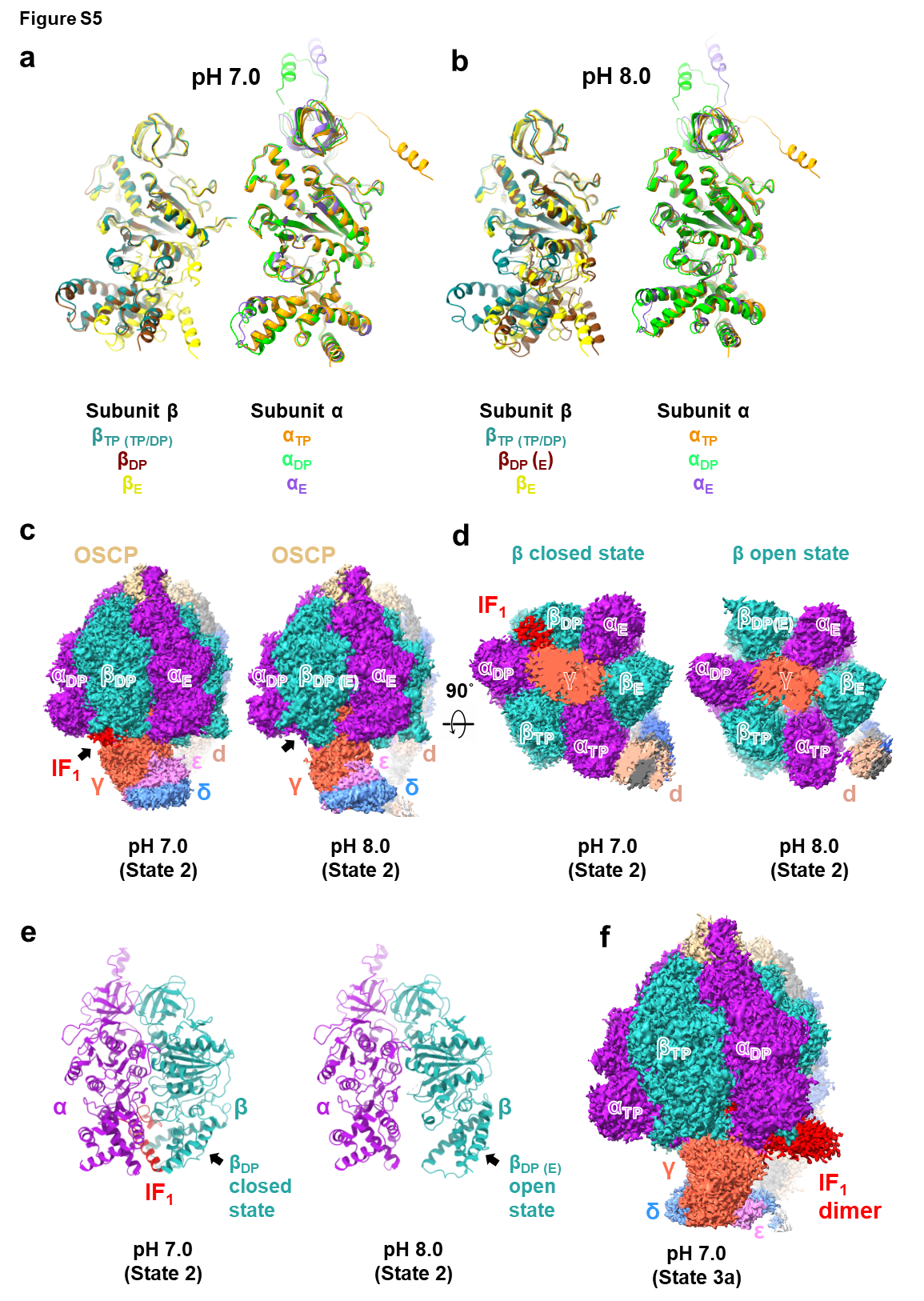


**Figure S5. Structural differences in *A. franciscana* ATP synthase F_1_ domain at pH values 7.0 and 8.0.** **a**, "Walker-Boyer" state geometries are observed for *A. franciscana* α and β subunits at pH 7.0 and **b**, pH 8.0, with "open" conformation (β_E_, "empty") and "loose" conformation (β_TP/DP_ with bound adenosine 5'-diphosphate or adenosine 5’-triphosphate). **c,** side views and **d,** bottom views of the F_1_ local refined cryo-EM maps of *A. franciscana* ATP synthase (rotational state 2) at pH 7.0 and 8.0. An extra density corresponding to the IF1 monomer is present in the map obtained at pH 7.0 but absent at pH 8.0. **e**, The β subunit is found in the closed state in the presence of IF1 (pH 7.0), while it is in the open state in the absence of IF1 (pH 8.0). **f**, IF1 dimer density was found in F_1_ focused refined map of rotational state 3a at pH 7.0.


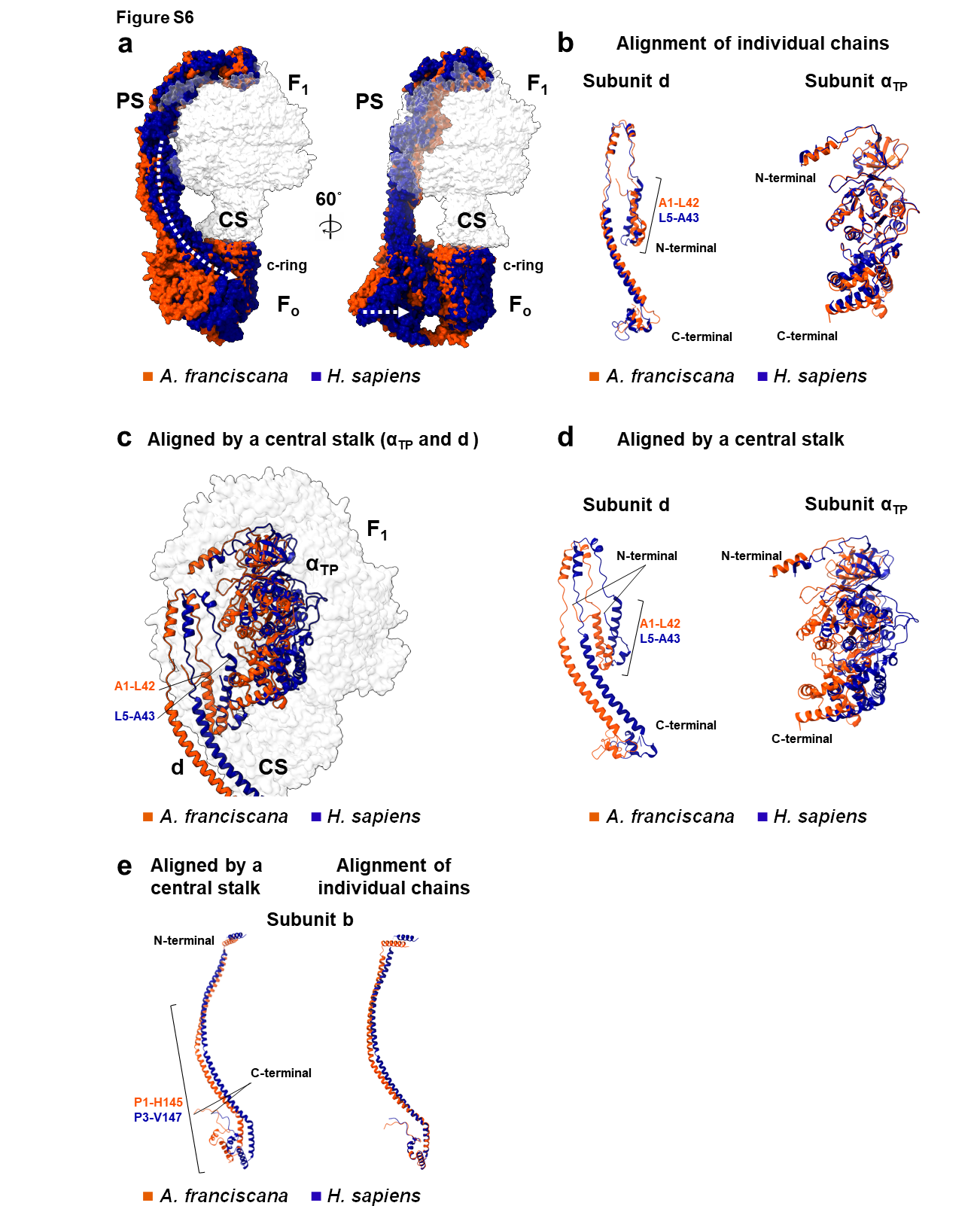


**Figure S6. Structural differences in the peripheral stalk subunits of *A. franciscana* and *H. sapiens* ATP synthase. a.** Surface representation of the ATP synthase models of *A. franciscana* and *H. sapiens*, aligned by the central stalk subunits. The peripheral stalks and F_O_ domains are shown in orange and blue in *A. franciscana* and *H. sapiens* structures, respectively. The white dashed arrows indicate the shift of the peripheral stalk and F_O_ in *H. sapiens* compared to *A. franciscana*. **b.** Individual alignment of d-subunits shows less apparent differences between the segments A1-L42 (*A. franciscana*) and L5-A43 (*H. sapiens*). The individual alignment of α_TP_ subunits shows no differences in the two structures. **c.,** **d.** The comparison of subunits d and α_TP_ shows a shift in human subunits towards the central stalk. In contrast, α_TP_ is moved towards the d-subunit in *A. franciscana*, leading to more enhanced interactions between these subunits described in Fig. S7. The α-helical segment of the *A. franciscana* d-subunit (dA1-dL42) has more upright conformation than the corresponding segment in the human d-subunit (dL5-dA43), which is tilted towards α_TP_ and CS. **e.** The comparison of *A. franciscana* and *H. sapiens* b subunit structures. Subunits are aligned either by the central stalk or individually. Differences are noticed mainly in the *A. franciscana* b-subunit segment consisting of residues P1-H145 compared with the corresponding segment, P3-V147, in *H. sapiens*. State 2 models of *A. franciscana* (PDB:9B0X) and *H. sapiens* (PDB:8H9T) ATP synthase were used in all panels.


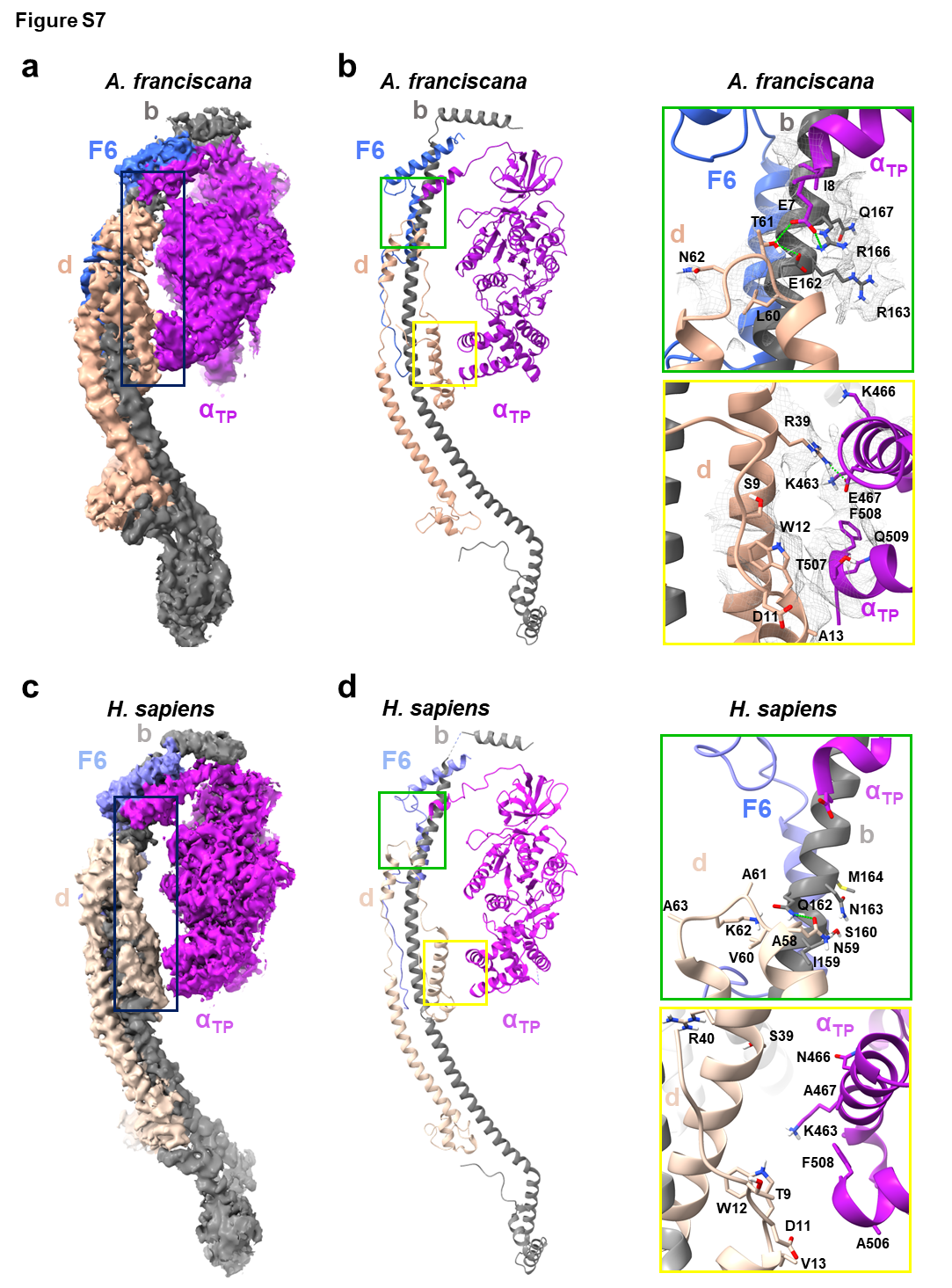


**Figure S7. Structural analysis of *A. franciscana* and *H. sapiens* ATP synthase peripheral stalk subunits b, d and their interactions with α_TP_. a.** The cryo-EM map of *A. franciscana* ATP synthase showing interactions between subunits α_TP_, F6, b, and d. The black box highlights the proximity of subunits α_TP_ and d. **b.** The cartoon representation of the subunits α_TP_, F6, b, and d in *A. franciscana*. Potential interactions between the α_TP_ N-terminal domain with subunits d and b are shown in the green box. A hydrogen bond may form between dT61 and α_TP_ E7 (d=3.8Å) and dT61 and bE162 (d=3.2 Å). A salt bridge may form between α_TP_ E7 and bR166 (d=3.6 Å). The potential interactions between subunit d and the C-terminal region of α_TP_ are shown in the yellow box. A salt bridge may form between the dR39 and αE467 (d=3.3Å)*.* **c.** The cryo-EM map of *H. sapiens* ATP synthase shows interactions between subunits α_TP_, F6, b, and d. The black box highlights the farther distance between subunits α_TP_ and d in *H. sapiens* compared with the *A. franciscana* structure shown in (**a**). **d.** The cartoon representation of the subunits α_TP_, F6, b, and d in *H. sapiens*. There are fewer interactions in human structures compared with *A. franciscana.* The interactions between the N-and C-terminal domains of the α_TP_ with d observed in *A. franciscana* are absent in the human structure (green and yellow boxes). A hydrogen bond may form between the dN59 and bQ162 (green box). For clarity, other ATP synthase subunits were omitted from the figure. All hydrogen bond and salt bridges are shown with green dotted lines. State 2 models of *A. franciscana* (PDB:9B0X) and *H. sapiens* (PDB:8H9T) ATP synthase were used in all panels.


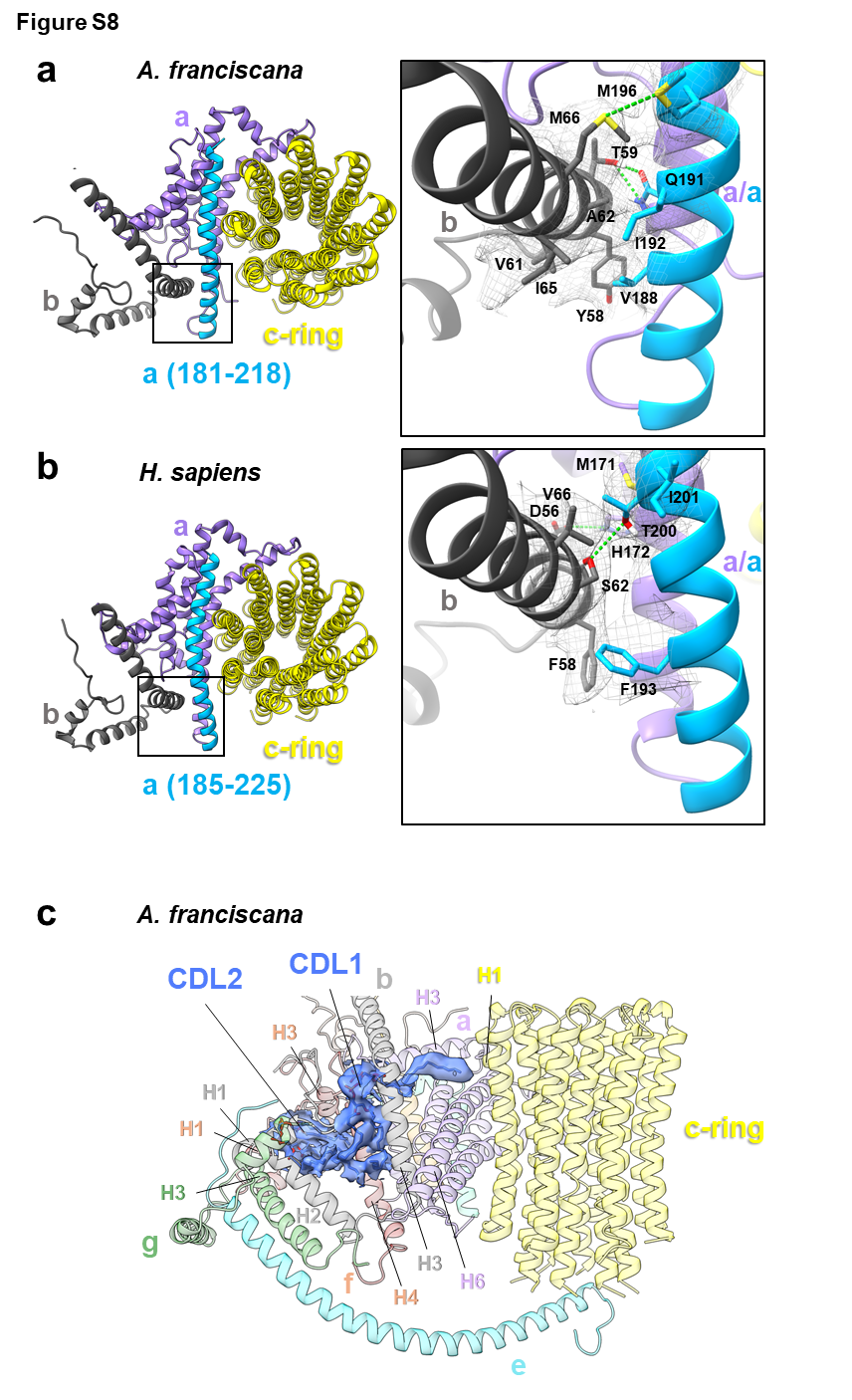


**Figure S8. Structural analysis of *A. franciscana* and *H. sapiens* ATP synthase a and b subunits. a.** The top view of the *A. franciscana* F_O_ domain shows interactions between subunits a (purple and blue), b (grey), and c (yellow). The α-helical segment of a-subunit (residues 181-218, colored blue) shows a more linear conformation compared with the corresponding sequence in *H. sapiens*. A disulfide bond may form between bM66 and aM196 (d= 3.9Å), and two hydrogen bonds may form between the hydroxyl group of bT59 with the carbonyl and the amine group of aQ191 (d= 2.4Å and d=3.4Å, respectively). **b.** The α-helical segment of a-subunit (residues 185-225, colored blue) has a more curved conformation in *H. sapiens*. The following interactions were found between subunits a and b: A hydrogen bond between bS62 and aT200 and a salt bridge between bD56 and aH172. All hydrogen bond and salt bridges are shown with green dotted lines. **c.** Cartoon representation of *A. franciscana* ATP synthase F_O_ domain shows two cardiolipin molecules (CDL1 and CDL2) fitted into their respective densities in the map. CDL1 is located between the subunits a, b, and f, interacting with the α-helical domains aH3, aH6, cH1, bH3, and fH4. The second cardiolipin molecule, CDL2, was found between the subunits b, f, and g and interacts with the helical domains fH1, fH3, fH4, bH1, bH2, and gH3. State 2 models of *A. franciscana* (PDB:9B0X) and *H. sapiens* (PDB:8H9T) ATP synthases were used in all panels.


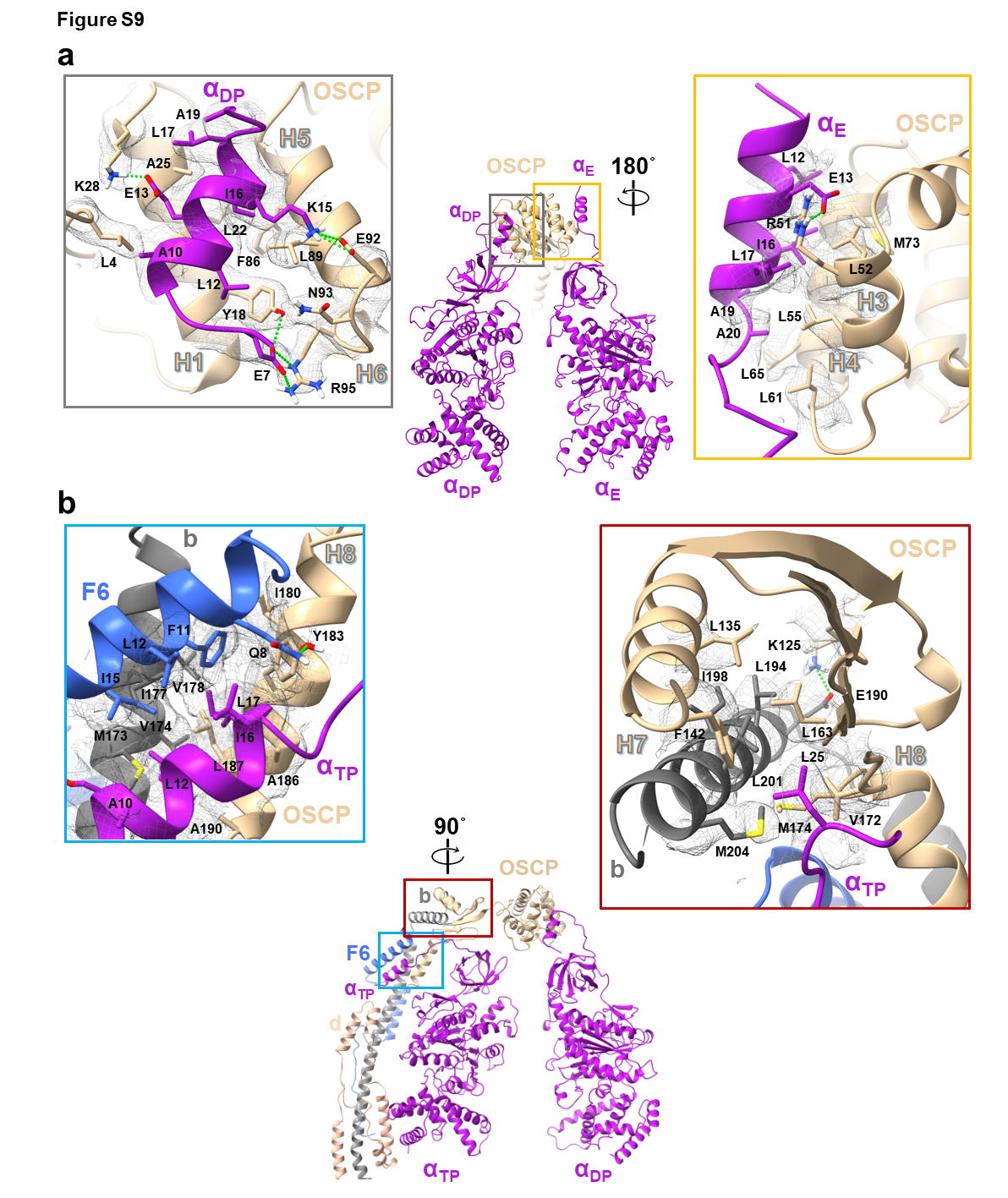


**Figure S9. Interactions between the N-terminal regions of α-subunits with OSCP in *A. franciscana* ATP synthase. a,** Left panel in the grey box: Interactions between the α_DP_-subunit and H1 and H5 helices of OSCP. Salt bridges may form between αK15 and OSCP E92 (d=2.8Å). Two salt bridges may form between αE7 and OSCP R95 (d=2.5Å and d=3.3Å). A salt bridge and a hydrogen bond may form between the αE13 and OSCP H1K28 (d=2.6Å) and αE7 and H1Y18 (d=2.8Å), respectively. The right panel in the orange box shows interactions between the α_E_-subunit with the H3 and H4 helices of OSCP. A salt bridge may form between the αE13 and OSCPR51 residues (d=3.7Å). The middle panel is a zoomed-out view of the α_DP_ and α_E_ interactions with the OSCP. **b,** The left panel in the light blue box shows interactions between the α_TP_ and the peripheral stalk subunits d, b, F6, and C-terminal domain of OSCP (H8 helix). The α_TP_-subunit interacts with F6 through a hydrogen bond between αS9 and F6D19 (d=2.5Å). The α_TP_-subunit interacts with subunit d through a hydrogen bond between αE7 and dT61 (d=3.8Å) and with subunit b through a salt bridge between αE7 and bR166 (d=3.5Å). Another hydrogen bond and a π-π stacking interaction may form between the OSCP Y183 and F6 Q8 (d=3.0Å) and the OSCP Y183 and F6 F11 (d<5.0Å), respectively. The right panel in the red box shows interactions between the α_TP_, F6, b, and OSCP H7 and H8 helices. A salt bridge may form between bE190 and OSCP K125 (d=2.6Å). The side chains of non-polar residues involved in hydrophobic interactions are shown. The middle panel is a zoomed-out view of the α_TP_ interactions with the OSCP and other peripheral stalk subunits. The green dotted lines show all the salt bridges and hydrogen bonds.


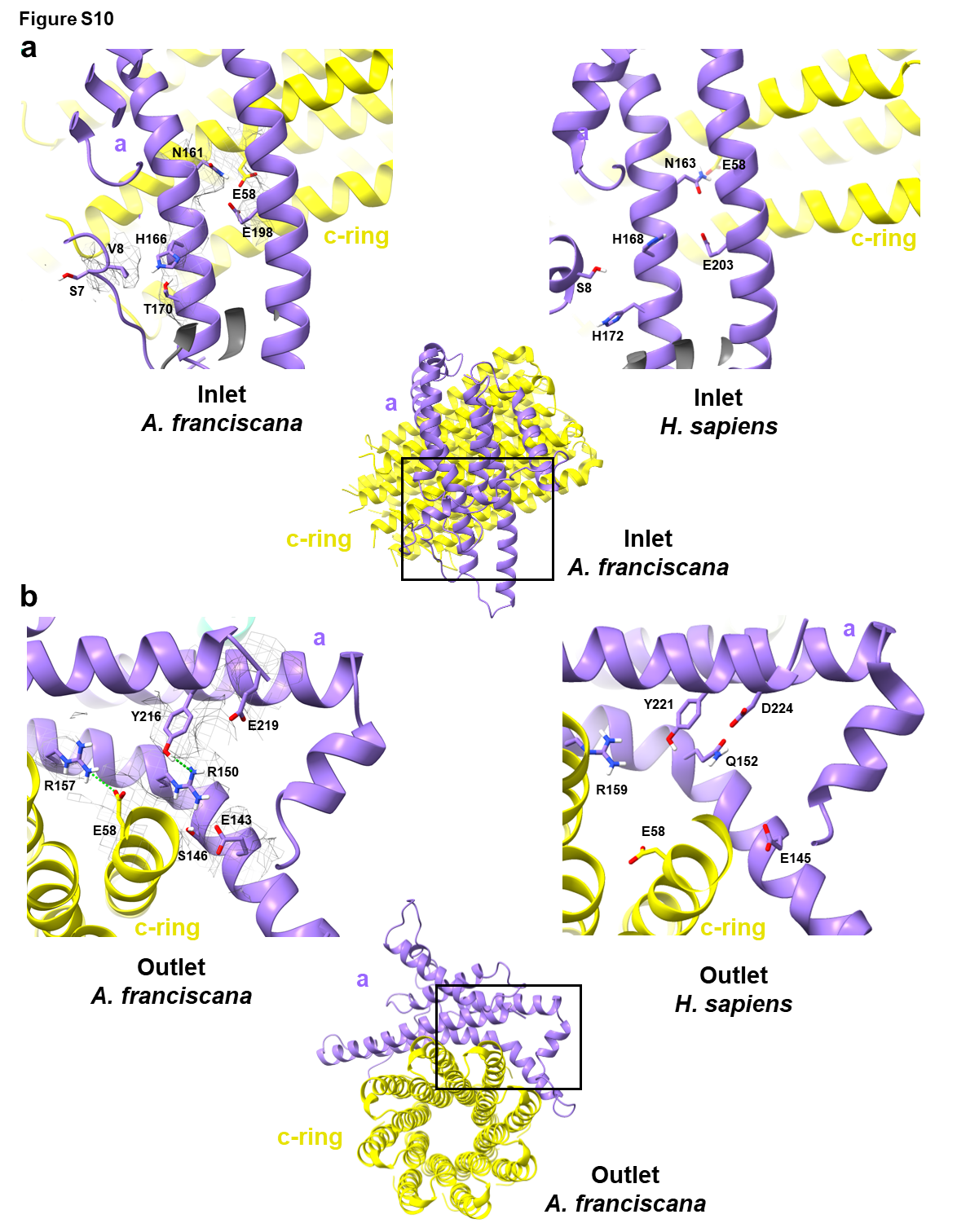


**Figure S10. Proton translocation pathway in *A. franciscana* F_O_**. **a,** Left and right panels, inlet half-channels of *A. franciscana* (PDB:9B0X) and *H. sapiens* (PDB:8H9T) a-subunits, respectively. The middle panel is a zoomed-out view of the inlet channel at the interface of the a- and c-subunits. **b,** Left and right panels, outlet half-channels of *A. franciscana* and human a-subunits, respectively. The middle panel is a zoomed-out view of the outlet channel at the interface of the a- and c-subunits. Key residues of the a-subunit and the c-ring are indicated. The green dotted lines indicate the possible formation of salt bridges between the conserved residues cE58 and aR157 (d=2.6Å), aR150 and aY216 (d=3.6Å) in *A. franciscana*.


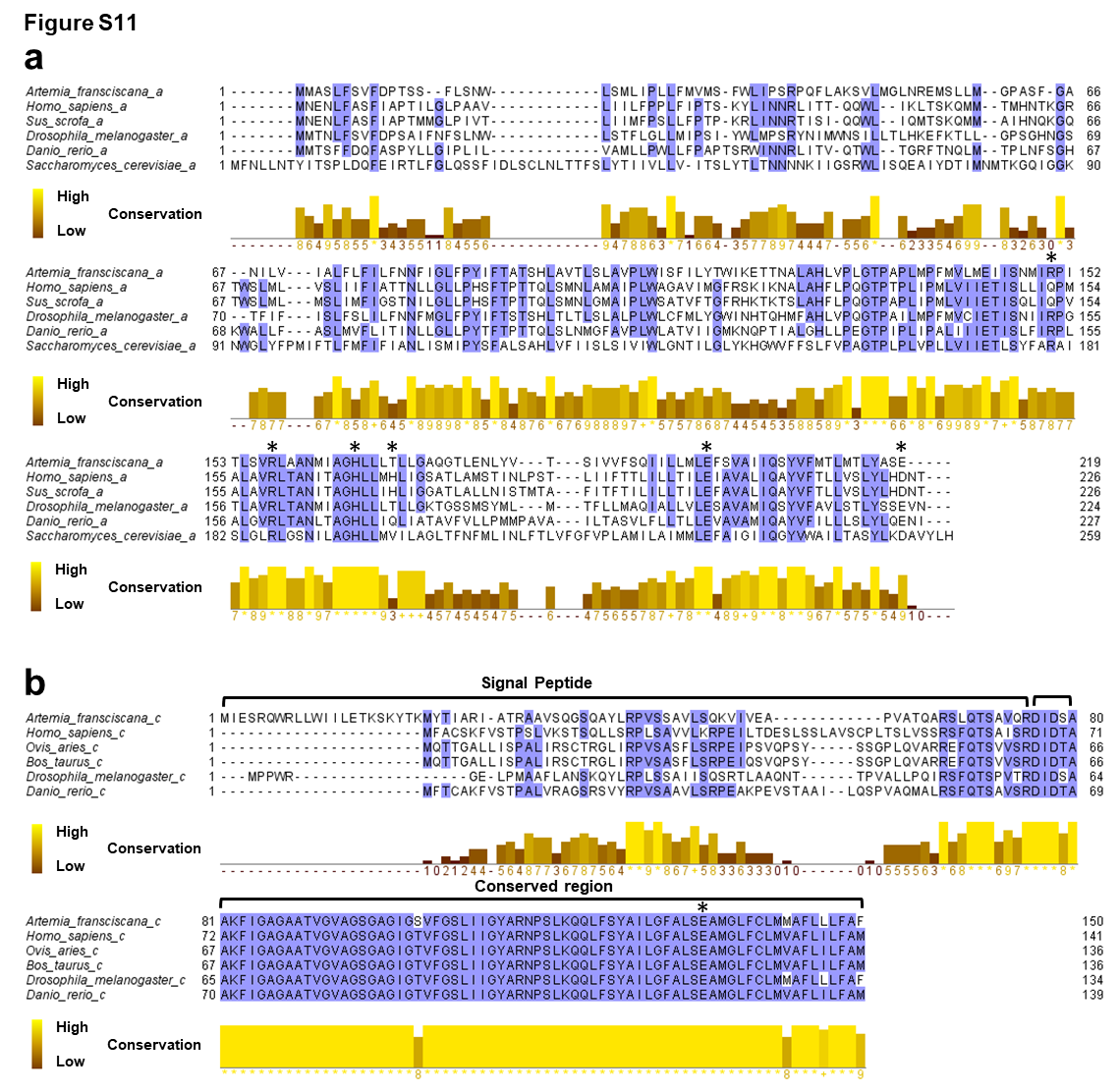


**Figure S11. The amino acid sequence alignments of subunits a and c in different species show higher conservation of the c- but not a-subunit sequences.** **a.** The amino acid sequence alignment of a-subunit was generated through ClustalW Omega and treated using the BLOSUM62 color scheme in JalView, highlighting residues with an identity above 60%. Two barrier arginine residues, R150 and R157 that separate the inlet and outlet half-channels of the a-subunit are indicated with asterisks. The inlet half-channel of a-subunit at the inner membrane starts with the aH168 and aH172 residues in mammals^46, 67^. The aH168 is conserved in *A. franciscana* (aH166), while the aH172 is replaced with threonine (aT170). Residues aH166 and aT170 are marked with asterisks. The key direct proton donor/acceptor glutamates, E198 in the inlet and E219 in the outlet channels are also shown with asterisks. **b.** The amino acid sequence alignment of the c-subunit was generated through ClustalW Omega and treated using the BLOSUM62 color scheme in JalView, highlighting residues with an identity above 60%. The conserved cE58 residue is marked by an asterisk.


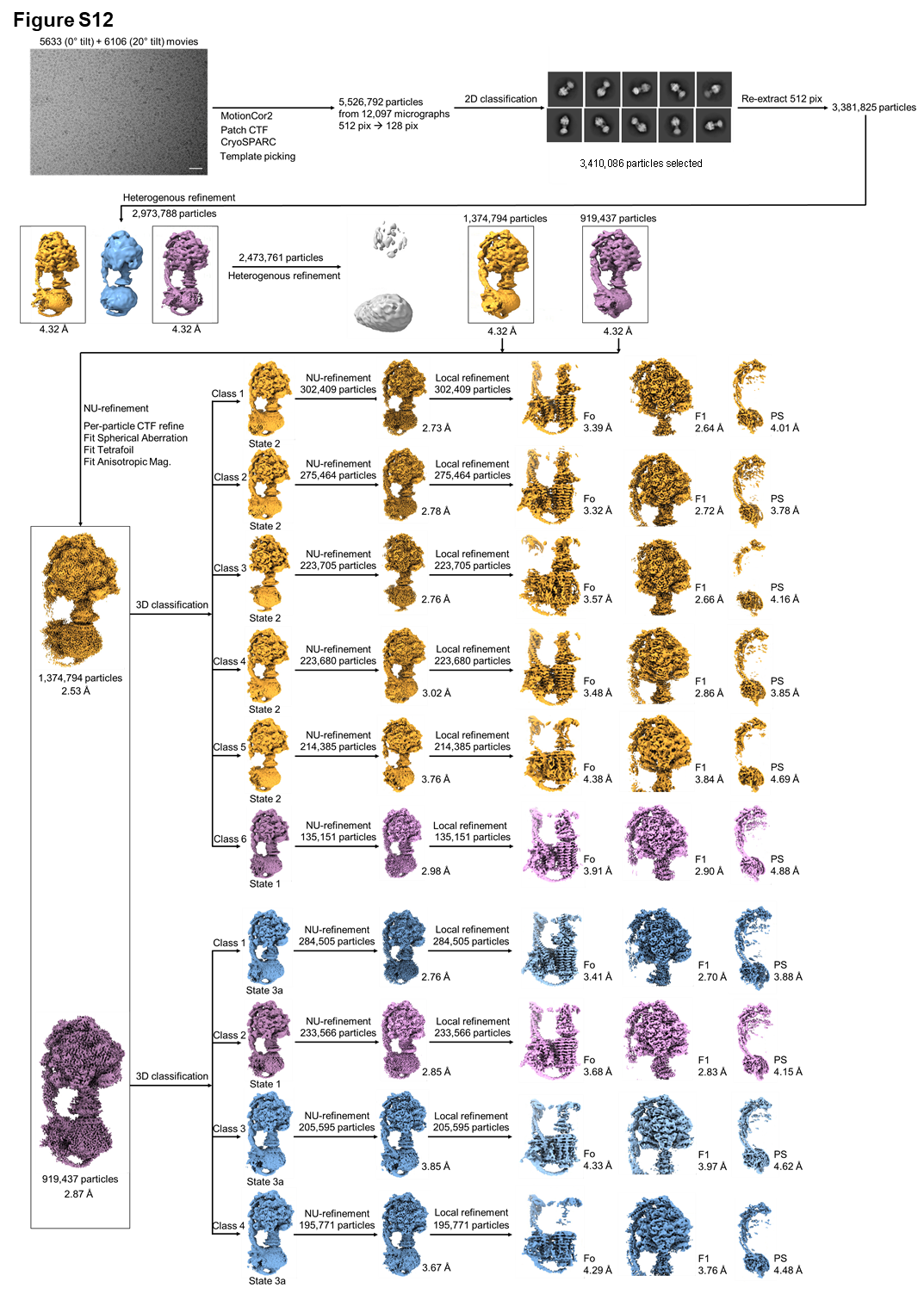


**Figure S12.** **Cryo-EM data processing of *A. franciscana* ATP synthase purified at pH 8.0.** The data processing flowchart of *A. franciscana* ATP synthase in three rotational states, state 1, state 2, and state 3a. The focused refinement of the local F_O_, F_1_, and Peripheral Stalk (PS) regions in three rotational states was performed. Scale bar, 20 nm.


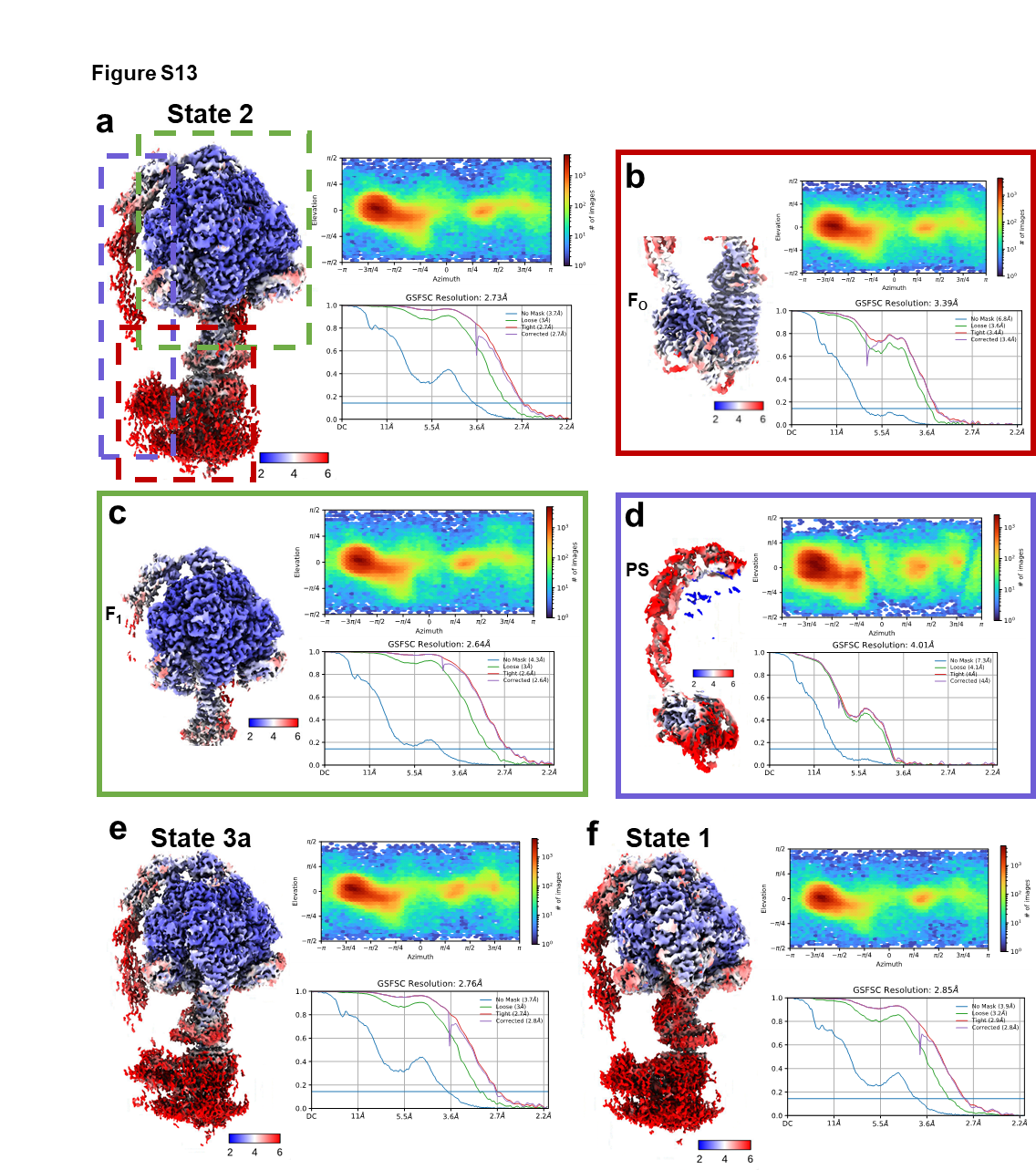


**Figure S13**. **Local resolution estimation, particle distribution, and Gold Standard Fourier Shell Correlation (GSFSC) resolution estimation of the *A. franciscana* ATP synthase (pH 8.0) in different rotational states**. **a**, State 2. **b**, State 2 F_O_ local-refined, **c**, State 2 F_1_ local-refined, **d**, State 2 PS local-refined, **e**, State 3a. **f**, State 1.


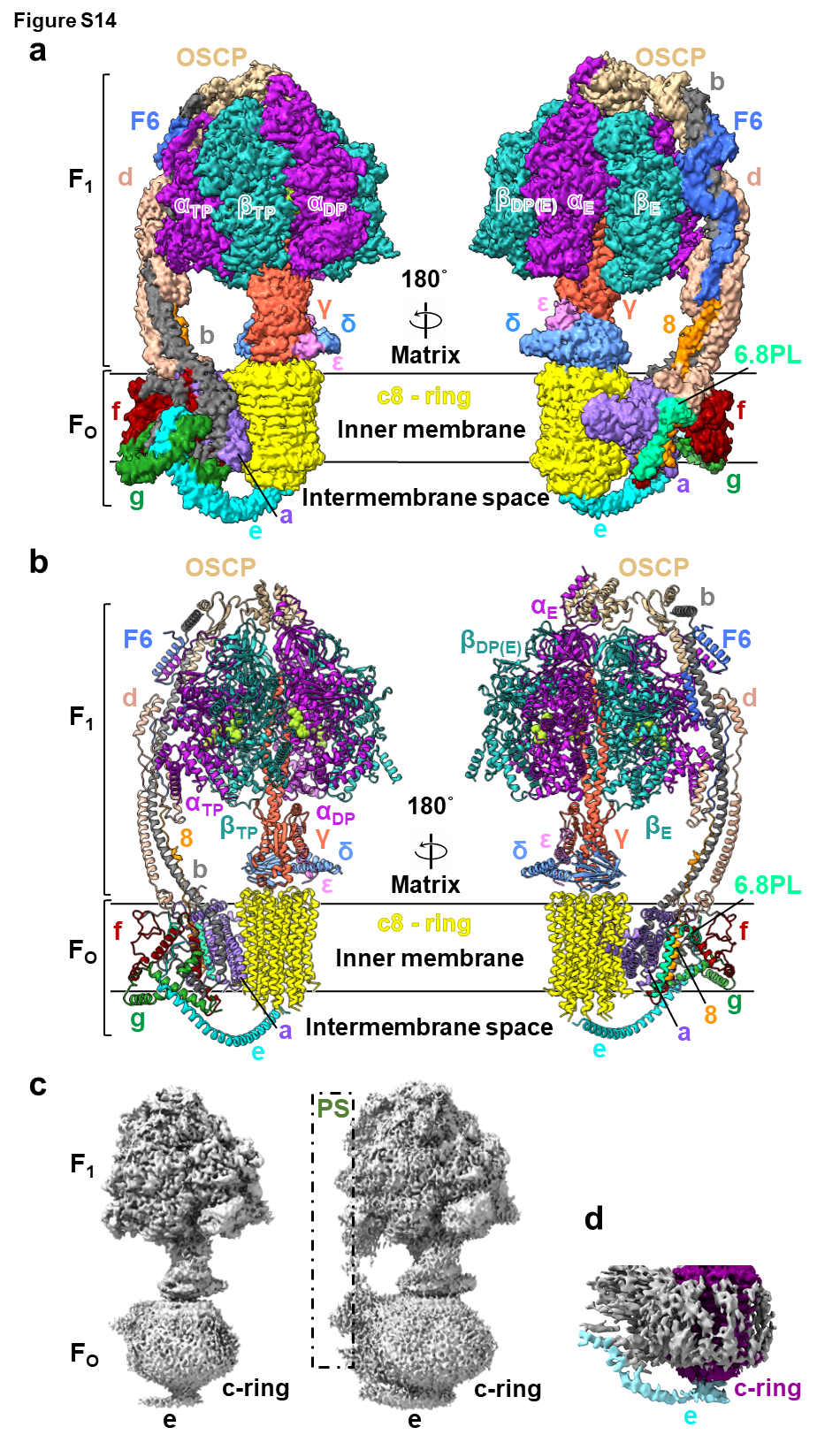


**Figure S14. The overall structure of *A. franciscana* ATP synthase, purified at pH 8.0.** **a**, Composite map of *A. franciscana* ATP synthase in state 2 shown is surface representation. **b**, Cartoon representation of the atomic model of *A. franciscana* ATP synthase subunits. **c,** Cryo-EM map of the *A. franciscana* ATP synthase in the state 2 (Class 3, 223,705 particles) at pH 8.0 shown in two different threshold levels to highlight the absence of clear and complete densities for the peripheral stalk (PS) and subunits a, g, f, and 6.8PL of F_O_ at pH 8.0. **d**, The F_O_ local refined map of the same state 2 (Class 3) shown in **(c)**, demonstrates a well-defined density for subunit e and its interaction with the c-ring in the absence of apparent density of peripheral stalk.

| **Table S1. Cryo-EM data collection, refinement, and validation statistics** | | | | | | | | |
| --- | --- | --- | --- | --- | --- | --- | --- | --- |
| **Parameters** | ***A. franciscana* ATP synthase, pH 7.0** | | | | | | | |
|  | State 1, F_O_F_1_  (EMD-44087) | State 2, F_O_F_1_, consensus map  (EMD-49580) | State 2, F_O_F_1_, composite structure  (EMD-44061  PDB:9B0X) | State 3a, F_O_F_1_ (EMD-44094) | State 3a,  local-refined F_1_ (EMD-44096) | State 2,  local-refined F_O_ (EMD-44162) | State 2,  local-refined F_1_ (EMD-44165) | State 1,  local-refined F_O_ (EMD-44776  PDB:9BPG) |
| Microscope model | Titan Krios | | | | | | | |
| Direct detection camera | K3 | | | | | | | |
| Voltage (KeV) | 300 | | | | | | | |
| Nominal Magnification (kX) | 81 | | | | | | | |
| Pixel size (A^2^) | 1.068 | | | | | | | |
| Underfocus range (μm) | 1.0-1.7 (0 deg) and 0.7-1.3 (20 deg) | | | | | | | |
| Exposure rate  (e^-^/px/s) | 20.9 | | | | | | | |
| Total Dose (e^-^/Å^2^) | 56.88 | | | | | | | |
| Movies Collected | 6,440 (0° tilt) + 6,106 (20° tilt) | | | | | | | |
| Symmetry imposed | C1 | | | | | | | |
| Map resolution (Å) | 2.6 | 2.6 | 2.6 | 2.7 | 2.5 | 3.9 | 2.6 | 3.3 |
| FSC | 0.143 | 0.143 | 0.143 | 0.143 | 0.143 | 0.143 | 0.143 | 0.143 |
| **Refinement** | | | | | | | |  |
| Map sharpening B factor (Å) | 89.3 | 91.4 | 91.4 | 95.2 | 70.2 | 124.0 | 73.4 | 150 |
| **Model validation** | | | | | | | |  |
| MolProbity score | - | - | 1.84 | - | - | - | - | 2.12 |
| Clashscore | - | - | 4.93 | - | - | - | - | 5.66 |
| Poor rotamers (%) | - | - | 3.06 | - | - | - | - | 5.05 |
| **Ramachandran plot** | | | | | | | |  |
| Favored (%) | - | - | 96.65 | - | - | - | - | 95.92 |
| Outliers (%) | - | - | 0.02 | - | - | - | - | 0.00 |

| **Table S2.** | | |
| --- | --- | --- |
| **Chain name** | **Subunit name** | **Sequence origin** |
| 1 2 3 4 5 6 7 8 | Subunit C1 | *Artemia franciscana* |
| A B C | Subunit α | *Artemia franciscana* |
| D E F | Subunit β | *Artemia franciscana* |
| G | Subunit γ | *Artemia franciscana* |
| Q | protein 8 | *Artemia franciscana* |
| O | OSCP | *Artemia franciscana* |
| I | Subunit ε | *Artemia franciscana* |
| K | Subunit b | *Artemia franciscana* |
| L | coupling factor 6, F6 | *Artemia franciscana* |
| H | Subunit δ | *Artemia franciscana* |
| T | Subunit e | *Artemia franciscana* |
| R | Subunit f | *Artemia franciscana* |
| S | Subunit g | *Artemia franciscana* |
| P | Subunit 6.8PL | *Poly-A* |
| N | Subunit a | *Artemia franciscana* |
| M | Subunit d | *Artemia franciscana* |
| J | IF1 | *Danio rerio*/Uniprot |
| Ligand | ADENOSINE-5'-DIPHOSPHATE (ADP) | - |
| Ligand | ADENOSINE-5'-TRIPHOSPHATE (ATP) | - |
| Ligand | Cardiolipin (CDL) | - |
| Ligand | Magnesium ion (Mg) | - |

**Table S2. The list of the ATP synthase subunits, chains, and ligands found in the *A. franciscana* ATP synthase models.** The amino acid sequence information for most subunits was obtained through the *A. franciscana* genome annotation, excluding subunits IF1 and 6.8PL.

| **Table S3. Cryo-EM data collection, refinement, and validation statistics** | | | | | | | | |
| --- | --- | --- | --- | --- | --- | --- | --- | --- |
| **Parameters** | ***A. franciscana* ATP synthase, pH 8.0** | | | | | | | |
|  | State 1, F_O_F_1_  (EMD-44173) | State 2,  F_O_F_1_, consensus map  (EMD-49579) | State 2,  F_O_F_1_, composite structure  (EMD-44142  PDB:9B3J) | State 3a, F_O_F_1_  (EMD-43079) | State 2,  F_O_F_1_  with a weak density of the peripheral stalk (EMD-44177) | State 2, local-refined F_O_  (EMD-44169) | State 2, local-refined F_1_  (EMD-44170) | State 2, local-refined peripheral  stalk  (EMD-44172) |
| Microscope model | Titan Krios | | | | | | | |
| Direct detection camera | K3 | | | | | | | |
| Voltage (KeV) | 300 | | | | | | | |
| Nominal Magnification (kX) | 81 | | | | | | | |
| Pixel size (A^2^) | 1.068 | | | | | | | |
| Underfocus range (μm) | 1.0-1.7 (0 deg) and 0.7-1.5 (20 deg) | | | | | | | |
| Exposure rate  (e^-^/px/s) | 15.8 | | | | | | | |
| Total Dose (e^-^/Å^2^) | 58.52 | | | | | | | |
| Movies Collected | 5,633 (0° tilt) + 6,464 (20° tilt) | | | | | | | |
| Map resolution (Å) | 2.8 | 2.7 | 2.7 | 2.8 | 3.6 | 3.4 | 2.6 | 4.0 |
| FSC | 0.143 | 0.143 | 0.143 | 0.143 | 0.143 | 0.143 | 0.143 | 0.143 |
| **Refinement** | | | | | | | | |
| Map sharpening B factor (Å) | 78.1 | 79.4 | 79.4 | 80.1 | 78.9 | 92.7 | 74.1 | 110.9 |
| **Model validation** | | | | | | | | |
| MolProbity score | - | - | 1.70 | - | - | - | - | - |
| Clashscore | - | - | 4.19 | - | - | - | - | - |
| Poor rotamers (%) | - | - | 2.52 | - | - | - | - | - |
| **Ramachandran plot** | | | | | | | | |
| Favored (%) | - | - | 96.82 | - | - | - | - | - |
| Outliers (%) | - | - | 0.00 | - | - | - | - | - |

**Video S1. The three-dimensional view of *A. franciscana* (PDB:9B0X, state 2) and *H. sapiens* (PDB:8H9T, state 2) ATP synthase models aligned by the central stalk**. The white dashed arrows indicate the shift of the F_O_ and peripheral stalk subunits in *H. sapiens* (blue) compared to *A. franciscana* (orange). The peripheral stalk in *A. franciscana* displays a more relaxed conformation than in the human structure. The top view image shows that human α and β subunits are not shifted in the same direction along with the peripheral stalk and F_O_ compared to *A. franciscana* ATP synthase. The human α and β subunits are slightly rotated counterclockwise in relation to the *A. franciscana* α and β subunits when viewed from the top. The bottom view of compared structures shows a shift in the human F_O_ domain to the top and right when compared to *A. franciscana* F_O_.
