# Supplementary material for: Cryo-EM structure of the brine shrimp mitochondrial ATP synthase suggests an inactivation mechanism for the ATP synthase leak channel
Source: Cell Death Differ. 2025 Mar 19;32(8):1518–35. doi: 10.1038/s41418-025-01476-w (PMC12325954; doi:10.1038/s41418-025-01476-w)
Supplement: Supplementary file 5 — Video legend [file 41418_2025_1476_MOESM5_ESM.docx]

**Video legends**

**Video 1. The three-dimensional view of the *A. franciscana* ATP synthase (**PDB:9B0X, EMD-44061). Two salt bridges may form between residues K61 and D1 (d=4.0Å for both interactions). Two salt bridges may form between K79 and D1 (d=3.3Å and d=3.4Å). Two hydrogen bonds may form between the Y71 and D1 residues (d=2.5Å and 3.0Å). The residues K79 and Y71 may also interact with the lipids found inside the c-ring. The phosphatidylserine and lyso-phosphatidylserine were tentatively fitted into the densities of the c-ring lumen (matrix and intermembrane space sides, respectively) for visualization purposes only. The exact nature of the lipids occupying the *A. franciscana* c-ring remains to be identified.
